# Fine-Grained Topography and Modularity of the Macaque Frontal Pole Cortex Revealed by Anatomical Connectivity Profiles

**DOI:** 10.1007/s12264-020-00589-1

**Published:** 2020-10-27

**Authors:** Bin He, Long Cao, Xiaoluan Xia, Baogui Zhang, Dan Zhang, Bo You, Lingzhong Fan, Tianzi Jiang

**Affiliations:** 1 School of Mechanical and Power Engineering, Harbin University of Science and Technology, Harbin, 150080 China; 2 Brainnetome Center, Institute of Automation, Chinese Academy of Sciences, Beijing, 100190 China; 3 National Laboratory of Pattern Recognition, Institute of Automation, Chinese Academy of Sciences (CAS), Beijing, 100190 China; 4 Center for Excellence in Brain Science and Intelligence Technology, Institute of Automation, CAS, Beijing, 100190 China; 5 Key Laboratory for NeuroInformation of the Ministry of Education, School of Life Science and Technology, University of Electronic Science and Technology of China, Chengdu, 610054 China; 6 The Queensland Brain Institute, University of Queensland, Brisbane, QLD 4072 Australia; 7 University of CAS, Beijing, 100049 China; 8 College of Information and Computer, Taiyuan University of Technology, Taiyuan, 030600 China; 9 Chinese Institute for Brain Research, Beijing, 102206 China; 10 Core Facility, Center of Biomedical Analysis, Tsinghua University, Beijing, 100084 China

**Keywords:** Macaque, Frontal pole cortex, Anatomical connectivity profile, Parcellation, Neuroimaging, Principal component analysis

## Abstract

The frontal pole cortex (FPC) plays key roles in various higher-order functions and is highly developed in non-human primates. An essential missing piece of information is the detailed anatomical connections for finer parcellation of the macaque FPC than provided by the previous tracer results. This is important for understanding the functional architecture of the cerebral cortex. Here, combining cross-validation and principal component analysis, we formed a tractography-based parcellation scheme that applied a machine learning algorithm to divide the macaque FPC (2 males and 6 females) into eight subareas using high-resolution diffusion magnetic resonance imaging with the 9.4T Bruker system, and then revealed their subregional connections. Furthermore, we applied improved hierarchical clustering to the obtained parcels to probe the modular structure of the subregions, and found that the dorsolateral FPC, which contains an extension to the medial FPC, was mainly connected to regions of the default-mode network. The ventral FPC was mainly involved in the social-interaction network and the dorsal FPC in the metacognitive network. These results enhance our understanding of the anatomy and circuitry of the macaque brain, and contribute to FPC-related clinical research.

**Electronic supplementary material:**

The online version of this article (10.1007/s12264-020-00589-1) contains supplementary material, which is available to authorized users.

## Introduction

The macaque frontal pole cortex (FPC) has a homotypical cytoarchitecture and a location relative to other prefrontal regions that is similar to that of humans [1], which means that it has the potential to be an excellent model for understanding the mechanisms of the human brain [2, 3]. As a portion of the prefrontal cortex, this area that has undergone more extensive evolution [4], and it is a late-developing area of the neocortex [5]. The FPC has a singularly high neuronal density and rich dendritic spines, which together suggest complex functions and multiple areas of cytoarchitectonic differentiation. In addition, the functional complexity of the FPC varies between species, which makes it a focal point for comparisons across species. Especially, as the core area involved in decision-making in the executive system [6, 7], the FPC has been pinpointed as a unique area that could separate humans from other primates with respect to higher cognitive powers [8, 9].

Although a variety of findings suggest that the macaque FPC can be divided into multiple functional subareas with different connectivity [10, 11], this area still lacks specialized research on its anatomical connections and a detailed parcellation map. Much of the previous work on this region primarily focused on cytoarchitecture and tract-tracing techniques [12–15]. The early researchers defined this area as Brodmann area (BA) 10 in humans and BA 12 in non-human primates [16]. Subsequently, this area was reclassified as BA 10 in non-human primates by Walker [17]. Recently, three primary studies have revealed markedly different cytoarchitectonic parcellation results [18–20], and many trace-injection experiments involving the FPC were based on previous rough maps of this area. In addition, the FPC has been suggested to be potentially associated with the default-mode network (DMN) [21, 22]. Statistical maps of enhanced activation have revealed that the FPC is involved in the social-interaction network (SIN) [23]. In macaque monkeys, Miyamoto, Setsuie, Osada, Miyashita [24] found that the FPC is recruited for the metacognitive judgment of non-experienced events by fMRI experiments. The inactivation of this area does not affect the detection of non-experienced events, but selectively impairs the metacognition of non-experienced events. From a different perspective, studies based on the diffusion tensor imaging (DTI) connectivity could further improve our understanding of the relationship between the macaque FPC and different functional networks, including the DMN, SIN, and metacognitive networks, but relevant studies are lacking. The above issues, which are both important and controversial, hint at the urgency of obtaining a detailed understanding of this region; however, to our knowledge, the macaque FPC remains one of the least understood brain areas [25]. Moreover, the traditional parcellation method based on cytoarchitectonics is not only limited to noninvasive approaches, but is also limited by the number of samples and lack of consideration of individual variation. Many trace-injection studies related to the FPC have been based on previous rough parcellation maps and relevant studies based on DTI are still a rarity.

In view of the importance of the diversity of functions of the monkey FPC and the lack of detailed anatomical connection information in comparison with the previous tracer results [19, 26, 27], as well as to pave the way for a systematic follow-up study using tracer injections, a study of the topological organization properties of the macaque FPC is necessary and attractive. Recently, connectivity-based parcellation (CBP) has been a powerful framework for mapping the human brain [28–30] and may provide a better picture of regional parcellation and anatomical connectivity information [31, 32] as well as allowing the target areas of tracer injections to be chosen less blindly. In this study, we provided a tractography-based parcellation scheme that applied a machine-learning algorithm to obtain a fine-grained subdivisions of the macaque FPC, and then revealed their subregional connections. Exploring the modular structure of a community and the anatomical connectivity patterns of different functional networks could help understand brain mechanisms and evolution, which contributes to FPC-related clinical research.

## Materials and Methods

**Fig. 1:**
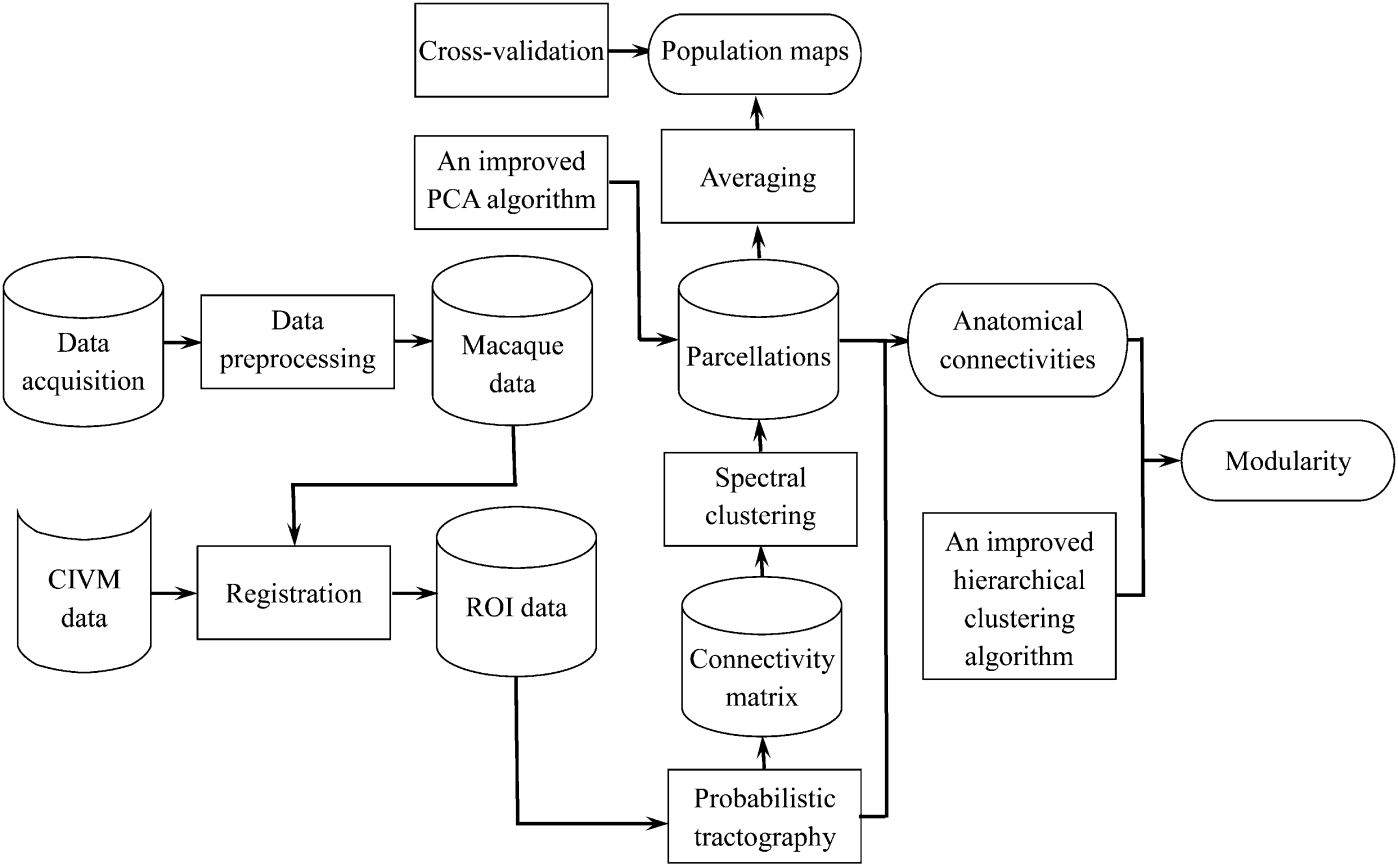
The overall workflow of this study.

To study the topological organization properties of the macaque FPC, three research objectives were established and the corresponding work was carried out. The overall workflow is shown in Fig. 1. First, we used DTI data to divide the macaque FPC into different subregions; then we explored the anatomical connectivity patterns of each subdivision. Furthermore, we proposed an improved hierarchical clustering algorithm to explore the modular structure of the community for the bilateral subregions.

### Macaque Brain Specimens

The rhesus macaques (*Macaca mulatta*) were obtained from Kunming Institute of Zoology, Chinese Academy of Sciences [33] (details in Table 1). All experimental procedures were conducted according to the policies set forth by the National Institutes of Health Guide for the Care and Use of Laboratory Animals, and approved by the Animal Care and Use Committee of the Institute of Automation, Chinese Academy of Sciences. They were judged by the veterinarian as appropriate subjects for euthanasia due to serious illnesses (acute gastroenteritis and enteritis). Each animal was intraperitoneally administered an overdose of pentobarbital [100 mg/kg, Sigma Aldrich (Shanghai) Trading Co., Ltd, Shanghai]. After verifying the status of deep anesthesia, they were transcardially perfused first with Phosphate-buffered saline (PBS) containing 1% heparin [pH 7.4, Sigma Aldrich (Shanghai) Trading Co., Ltd, Shanghai], followed by pre-cooled PBS containing 4% paraformaldehyde [Sigma Aldrich (Shanghai) Trading Co., Ltd, Shanghai]. Five minutes after starting the perfusion, the rate was lowered to 1 mL/min from an initial rate of 20 mL/min, and the entire perfusion lasted 2 h. The head was then removed and stored in PBS containing 4% paraformaldehyde. Then, the skull was carefully removed to expose the whole brain for MRI scanning. No apparent structural anomalies were found in any of the brains used in the present study.

**Table 1:** Information about the eight monkey brains.

Perfusion date	Number	Gender	Age	Weight (kg)
2016/05/09	93310	Female	23	3.24
	08046	Female	8	3.58
	12027	Male	4	3.06
	12411	Male	4	2.89
2016/05/10	01006	Female	15	3.57
	04084	Female	12	4.23
	10427	Female	6	3.69
	11402	Female	5	2.9

### MRI Acquisition

All the macaque MRI data were obtained using a 9.4T horizontal animal MRI system [Bruker Biospec 94/30 USR, with Paravision 6.0.1 (Ettlingen,Baden-Württemberg, Germany)]. Radiofrequency (RF) transmission and reception were achieved with a 154-mm inner-diameter quadrature RF coil. The SpinEcho DTI sequence used for the DTI data provided the main parameters: 74 slices, echo time (TE) = 22 ms, repetition time (TR) = 9800 ms, field of view (FOV) = 94 × 66 mm^2^, flip angle (FA) = 90°, acquisition matrix = 140 × 110, and resolution = 0.6 × 0.6 × 0.6 mm^3^ without gap. This sequence produced a complete set of 64 images, including 4 non-diffusion-weighted images (b = 0 s/mm^2^) and 60 images with non-collinear diffusion gradients (b = 1000 s/mm^2^) and required ~115 h of scanning time per specimen. T1-weighted data were acquired using a 2D IR-prepared RARE sequence with these main parameters: 74 slices, TE = 5.8 ms, TR = 4019 ms, inversion time = 750 ms, matrix = 280 × 220, FA = 90°, resolution = 0.3 × 0.6 × 0.3 mm^3^, FOV = 84 × 66 mm^2^, slice thickness = 0.6 mm, and no gap, requiring ~ 55 min. T2-weighted data were obtained in a 2D Turbo RARE sequence with these main parameters: 86 slices, TE = 30.9 ms, TR = 8464 ms, FA = 90°, resolution = 0.3 × 0.6 × 0.3 mm^3^, matrix = 280 × 220, FOV = 84 × 66 mm^2^, slice thickness = 0.6 mm, and no gap, requiring ~ 15 min.

### Definition of Seed and Target Masks of Macaque FPC

The macaque FPC seed masks were extracted from a publicly-available post-mortem macaque brain atlas (CIVM, https://scalablebrainatlas.incf.org/macaque/CBCetal15) [34] and all regional names were found in the list of abbreviations. This atlas is largely consistent with that of Paxinos *et al*. [18] Nissl-based atlas and has become increasingly popular in macaque studies [35–37]. The mask occupied the most rostral portions of the prefrontal cortex; its dorsal extent was bounded posteriorly by the anterior supraprincipal dimple (aspd) and did not cross the posterior supraprincipal dimple (pspd). In addition, on the medial aspect of the hemisphere, the FPC mask posteriorly bordered the cingulate sulcus (cgs) and in the coronal plane, it was ventrally delimited by the anterior termination of the olfactory sulcus. For each subject, the standard seed mask was wrapped back into individual diffusion space using the inverse of the deformations, and each resulting mask was visually inspected for possible errors and necessary modifications using ITK-SNAP (Philadelphia, Pennsylvania) [38]. To calculate the connectivity matrix and obtain the connectivity fingerprints, we extracted cortical regions and subcortical structures in the same hemisphere from the CIVM atlas as target regions [39]. The extraction approach for the target regions was the same as that for the FPC. Subsequently, we transformed them into individual diffusion space.

### Diffusion MRI Data Preprocessing

The diffusion MRI (dMRI) data were preprocessed using the FMRIB Diffusion Toolbox (FSL version 5.0; https://fsl.fmrib.ox.ac.uk/fsl/fslwiki/FSL), the prominent Medical Image Processing, Analysis, and Visualization software (MIPAV, https://mipav.cit.nih.gov/), and Advanced Normalization Tools (ANTs, http://www.picsl.upenn.edu/ANTS/), which is a state-of-the-art medical image registration toolkit [40]. The main procedure included the following steps. First, for the CIVM template, we transformed the raw format to the available dMRI space with MIPAV, which enables the quantitative analysis and visualization of medical images in different formats. Second, in the diffusion data from each subject, distortions caused by eddy currents were corrected using the FSL tool [41]. Finally, after conversion into the standard available format with MIPAV, the b0 image of the CIVM template space was co-registered to the individual non-diffusion-weighted images (b = 0 s/mm^2^) using ANTs. After the registration, an inverse transformation was performed to transform the seed and target masks for each subject’s small cortical areas into native dMRI space.

### Probabilistic Diffusion Tractography

After preprocessing, the macaque FPC was chosen as the seed and probabilistic tractography was carried out for the tractography-based parcellation. This process has been described in the toolbox for connectivity-based parcellation of the monkey brain [42] and is similar to that in another study [43]. Voxelwise estimates of the fiber orientation distribution were computed using Bedpostx. We calculated the probability distributions in two fiber directions at each voxel using a multiple fiber extension [44]. Based on the probability distributions, we then estimated the connectivity probability between each voxel in the seed region and every voxel of the whole brain using PROBTRACKX2 (Oxford, Oxfordshire). Probabilistic tractography was applied by sampling 15,000 streamline fibers per voxel and the step size was set to 0.2 mm [45, 46]. To exclude implausible pathways, we restricted how sharply pathways could turn, and the default threshold was set to 0.2. To correct the path distribution for the length of the pathways, we introduced a distance correction. The connection probability between each voxel in the seed region and any other voxel in the brain was obtained by computing the number of traces arriving at the target site. To decrease the number of false-positive connections, we thresholded the path distribution estimates for each subject using a connection probability value *P* < 20/15000 (20 out of 15,000 samples). Information about the connectivity was stored in an M-by-N matrix, where M denotes the number of voxels in the seed mask and N the number of voxels in the native diffusion space. To de-noise the data and increase computational efficiency, the connectivity profiles for each voxel were down-sampled to 2-mm isotropic voxels [47].

### Tractography-Based Parcellation of the Macaque FPC

Based on the connectivity patterns of all the voxels of the FPC, cross-correlation matrices were computed and fed into spectral clustering [28]. The maximum probability map (MPM) was computed by assigning each voxel of the standard space to the subarea in which it was most likely to be located [48]. First, we transformed the parcellation results from individual diffusion space to the CIVM template. Second, the MPM was computed according to the eight subjects’ parcellation results in CIVM space.

After parcellation of the FPC using spectral clustering, the next step was to select the number of clusters. To avoid an arbitrary choice of this number, we used two prevailing validation methods, cross-validation indices to obtain a consistent segmentation for all eight subjects at the group level, and principal component analysis (PCA), to determine the optimal number of clusters across the subjects at the individual diffusion level [42].

#### Cross-Validation Indices

The cross-validation offered two indices, Cramer’s V [49, 50] and topological distance (TpD) [51], for determining the optimal clustering number. Cramer’s V, is an indicator of clustering consistency and has values in the interval [0, 1], high values indicating good consistency. The TpD index, which quantifies the similarity of the topological arrangement of putative homologous regions in the bilateral hemispheres across all specimens, further determined the cluster number. The TpD score ranges from 0 to 1; a score close to 0 suggests that the two hemispheres have similar topology. The clustering number of local extremum points (peaks and valleys) means better consistency than that of adjacent ones, and in general, the local extrema are recommended as a good solution for each presumptive index [52, 53].

#### Principal Component Analysis

PCA, which requires no artificial hypothesis or prior knowledge, is a popular statistical framework for determining the clustering number [30, 54]. To ensure that the number of principal components to be chosen retain enough features and effectively represent the data, we proposed three criteria based on the literature [55, 56]. The first was the cumulative contribution, which means that a cumulative proportion of the variation could be explained by the eigenvalue obtained using the connection data. To obtain the cumulative proportion value (cpv), a threshold must be established. Generally, a sensible threshold is very often in the range 70% to 90%; it can sometimes be higher or lower depending on the practical details of a particular dataset. In our study, we thresholded the cpv at > 80%. Taking into account individual variation, we allowed a lower limit change of no more than 1%. The second criterion was that only factors with eigenvalues > 1 or next closest to 1 were retained. Specifically, the latter weak criterion (values close to 1) is like a “factorial scree” for atypical individual variation. The third criterion is a scree test [30, 54]. Briefly, for each subject, a ‘connectivity’ matrix between the various seeds and the whole brain was derived from the data of the probabilistic tractography. This matrix consisted of columns that indicated the FPC subregion of interest and rows that represented the whole-brain regions. To estimate the number of principal components to extract from each subject, a power curve was plotted by fitting the data, the inflexion point was extracted using a homemade routine written in MatLab (Natick, Massachusetts) R2017b, and all subjects were averaged to obtain a mean cluster value for the left and right hemispheres separately. Meanwhile, we set the difference threshold between the inflection point value of each individual and the average value as 0.5 to ensure that the clustering number among individuals was stable.

### Anatomical Connectivity Patterns

To explore the different anatomical connection patterns of the FPC subdivisions, we first drew 10^5^ samples from the fiber orientation distribution for each voxel in the subdivisions to calculate the whole-brain probabilistic fiber tracking [44]. To form the seed mask, each subarea was extracted from the probability map of the FPC at 25% probability. To reduce the false-positive rate and facilitate the qualitative analysis, we thresholded the connectivity probability value at 3.08 × 10^−5^ at the individual level, which means that at least 3.08 of the 10^5^ samples produced from each seed voxel were connected [48]. Next, the identified fiber tracts were binarized and transformed into CIVM macaque template space. All the binarized results were averaged to obtain population maps with a threshold of 50% [39], which means that only those voxels that were present in at least 4 of 8 subjects were mapped, and were then transformed into F99 space for display.

Subsequently, to visualize the differences in the anatomical connectivity of each subarea, we further calculated the anatomical connectivity fingerprints between each subarea and each of the target regions in the CIVM atlas. For the eight subjects, these target brain regions, including the cortical areas and subcortical structures of each subject, were extracted from the CIVM atlas in the same hemisphere using the same method as used to extract the FPC and was subsequently transformed into individual dMRI space. Using their fingerprints, we were able to find the different connectivity properties for each subregion. In addition, we performed similarity analysis of the connections for these clusters to estimate the connection similarity between individuals. Briefly, we computed the correlation coefficients for the seed-to-target connections and obtained the *P* values for the hypothesis that there was no relationship between the observed phenomena. We defined the threshold of statistical significance as statistically highly significant at *P *< 0.001.

Furthermore, we also investigated and summarized other tract-tracing studies involving the macaque FPC to compare their consistency by assessing the repeatability of the connected areas that they identified. As a preliminary qualitative comparison, we collected the regions connected to the FPC from the CoCoMac database [57] and compared them with the regions connected to subdivisions of the FPC. The CoCoMac database provides convincing structural connectivity data for the macaque brain and was a remarkable effort by many researchers. Currently, it is the largest macaque connectivity study, with data extracted from > 400 published tract-tracing studies of the macaque brain. We also made a further comparison with some of the detailed trace-injection experiments.

Moreover, inspired by a human frontal pole study [50], to reveal a clearer picture of different functional networks from the perspective of anatomical connections, we analyzed the connections between the subregions and the regions of different functional networks. Specifically, we combined the regions that were connected to the DMN [21, 22], SIN [23], and metacognition network [24, 58] and estimated the linkages and differences between the subareas. Additional comparisons with other studies and findings are presented in the Discussion.

### Mapping the Hierarchical Module Structure for FPC Subregions

Exploring the modular structure of a community and the connectivity patterns of different functional networks is important for understanding brain mechanisms and evolution. Many neuroscientific studies [59–61] have revealed that the brain networks share important organizational principles in common, such as modularity, and that topological modules often comprise anatomically neighboring cortical areas. In addition, the modules of brain networks contain both unilateral and bilateral areas [62], and a community structure in the brain can be correlated with functionally localized regions, such as visual, auditory, and central modules [63]. Here, we proposed an improved hierarchical clustering algorithm to examine the subregional members of the bilateral FPC and assign them to clusters. The correlation coefficient matrix of the native connectivity data was fed into the algorithm; as the number of clusters increased, those with high connection similarity were given priority and grouped together. Besides, we calculated the cophenetic correlation coefficient to evaluate and select the optimal clustering scheme. The hierarchical clustering method not only uncovered the modular community structure of the bilateral FPC, but also provided some methods that other researchers can use in making within-species comparisons. In particular, this method can be used for comparisons between parcellations with a greater number of subdivisions and those with fewer subdivisions that have been obtained from studies that use different methods. This approach can also be used to make heuristic comparisons between species, including comparisons of parcellation patterns and connectivity patterns involving different functional networks. Compared with previous studies [33, 43], the advantage of our method is that it is able to automatically select the optimal clustering by introducing the cophenetic correlation coefficient to compare the results of clustering the same data set using different distance calculation methods and clustering algorithms. The cophenetic correlation coefficient scores range from 0 to 1. The closer the value is to 1, the more accurately the clustering solution reflects the data.

## Results

### Connectivity-Based Parcellation and Subregional Anatomical Connectivity Patterns of the Macaque FPC

**Fig. 2:**
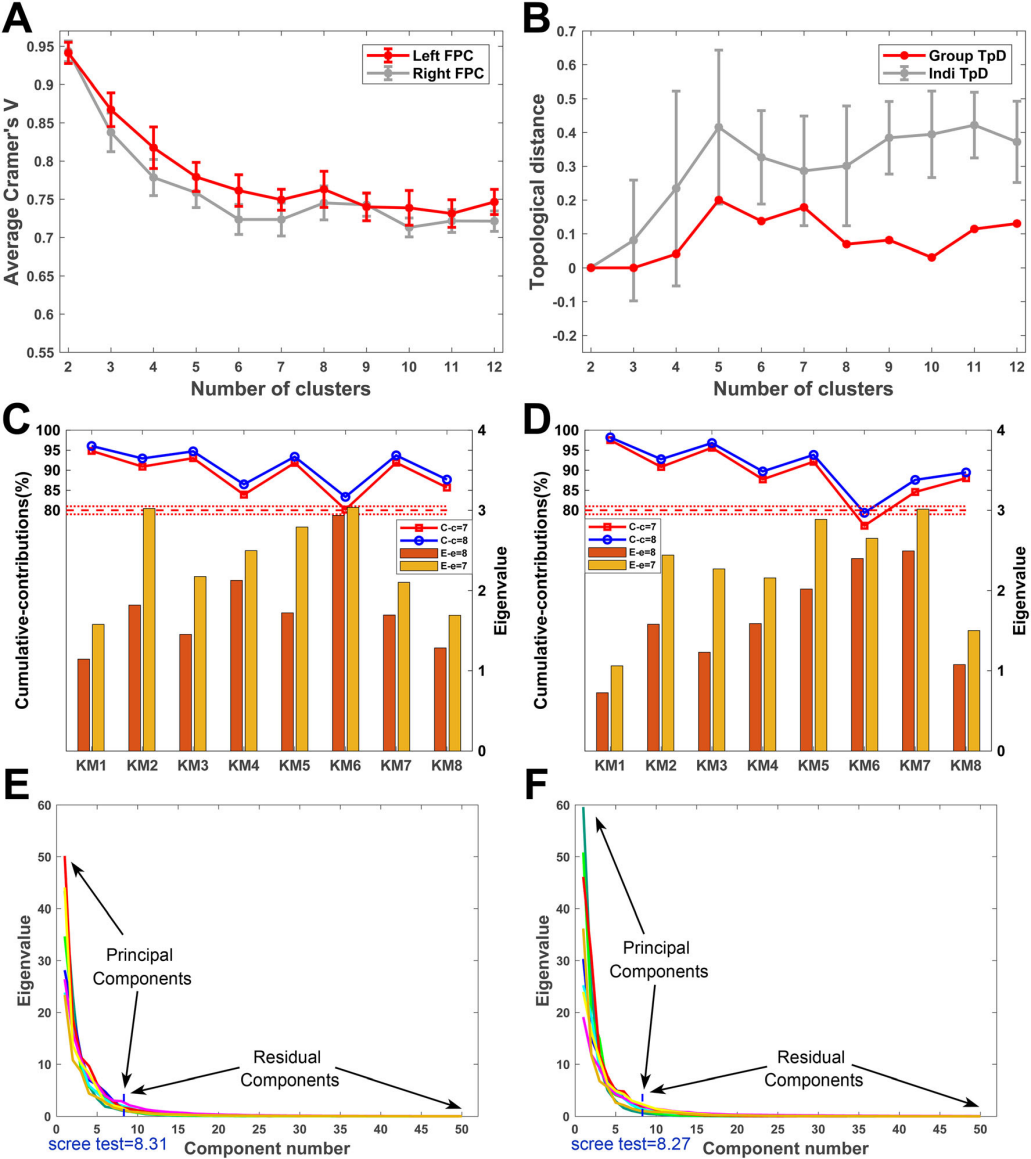
Cross-validity indices of parcellation of the FPC. **A**, **B** Cluster number consistency and topological similarity indicated by the average Cramer’s V (**A**) and TpD (**B**). The red/gray polyline of the average Cramer’s V indicates the clustering consistency of the left/right brain across subjects. The red/gray polyline of the TpD denotes the similarity of the topological arrangement of presumptive homologous regions between hemispheres and across subjects (KM1, KM2, …, KM8) at the group/individual level. **C**, **D** Cumulative contribution rates and eigenvalues for the left (**C**) and right (**D**) hemisphere under criterion 1 and criterion 2. A comparison of the blue and red curves reveals that eight principal components (blue) are superior to seven (red). The bars represent the eigenvalues of the seventh (yellow) and eighth (brown) components. **E**, **F** Graph of principal components according to their eigenvalue sizes for the left (**E**) and right (**F**) hemispheres for all specimens.

For the macaque FPC, the cross-validation of the spectral clustering data showed that the 8-cluster solution, as local extremum points of Cramer’s V, was optimal for a fine parcellation (Fig. 2A). The TpD index also selected the 8-cluster solution as optimal (Fig. 2B). These results of validity indices suggested the consistency of the parcellations across subjects and the similarity of the topological organization distribution of the parcellation results between the bilateral hemispheres at the group level [42]. In addition, at the individual diffusion space level, the index results of PCA also suggested a segregation of the FPC into an average of 8 subdivisions for each of the hemispheres (left hemisphere, 8.31, see Fig. 2E; right hemisphere, 8.27, see Fig. 2F).

**Fig. 3:**
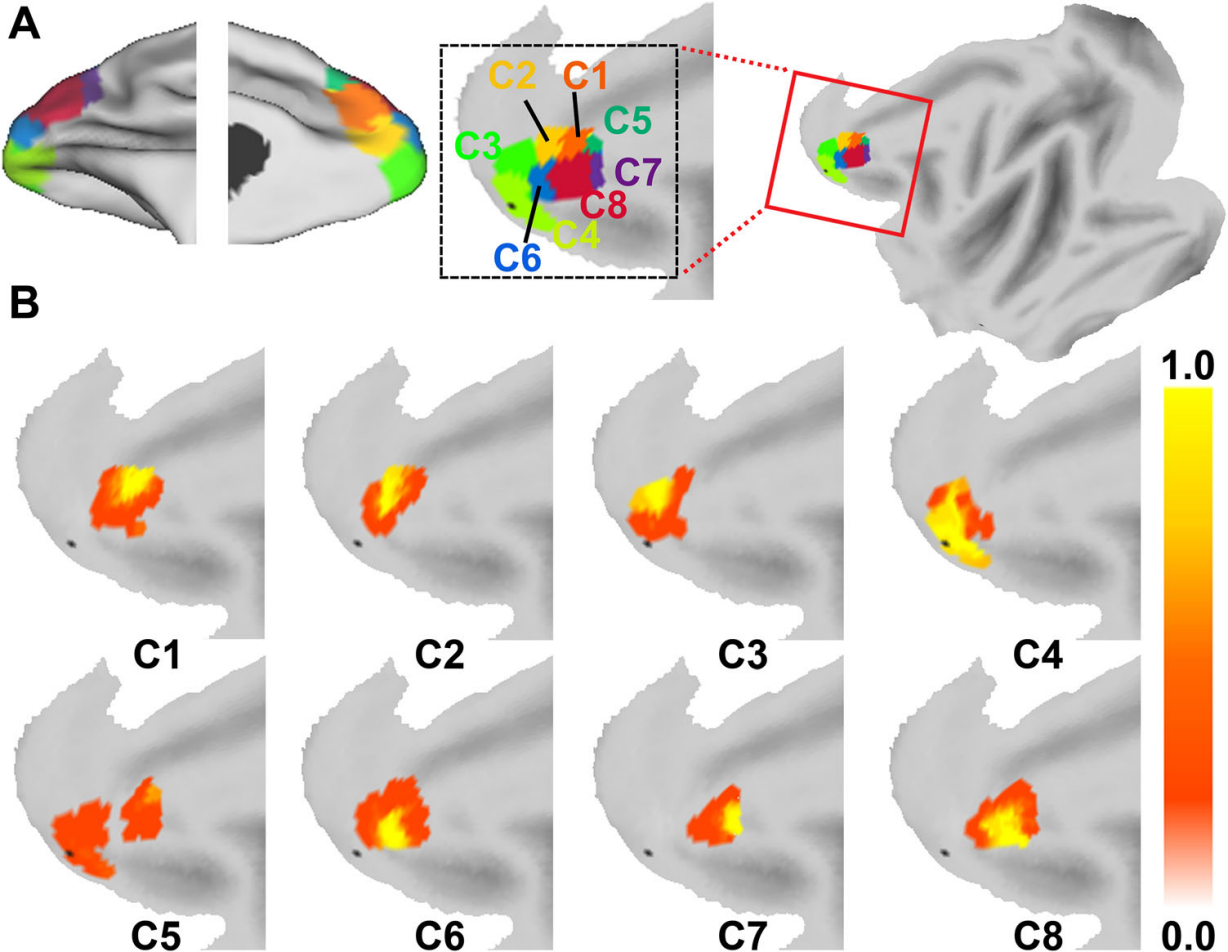
Connectivity-based parcellation (left hemisphere) of the macaque FPC on F99 surfaces. **A** The subdivisions are depicted on a flat surface (right) and a fiducial surface (left) of the lateral and medial views. Each subregion is coded with a unique color and named arbitrarily C1, C2, …, C8. **B** The probability map of each FPC subarea. The color bar represents the mean probability across subjects at each voxel.

The eight distinct subareas consisted of four components in the lateral section with the remaining four components in the medial section. These results were transformed and combined into F99 brain space [64] with Caret software [65] to create population-based parcellations of the FPC, and we further presented the probabilistic map for each subarea that could help to understand the consistency between subjects in the topography of the clusters (left hemisphere see Fig. 3; right hemisphere see Fig. S1). To facilitate understanding of the results, we determined the location of each subregion based on histologically defined cortical areas and a topologic map as well as on its anatomical connection information. In describing the location of these subregions, we refer to them with respect to the cgs, principal sulcus (ps), medial orbital sulcus (morbs), rostral sulcus (ros), aspd, and the adjacent areas. In the sagittal plane, a ventrolateral boundary along the direction of the ps separates C4 from C6, and another lateral boundary above the rostral ps distinguishes subareas C6 from C8. The dorsolateral boundary above the aspd separates subareas C8 from C7. In the medial FPC, a boundary along the anterior extension of the ros segregates subareas C3 from C2, and another boundary around the rostral cgs distinguishes subareas C1 from C2. Above the rostral cgs, a dorsomedial boundary separates subareas C5 from C1.

**Fig. 4:**
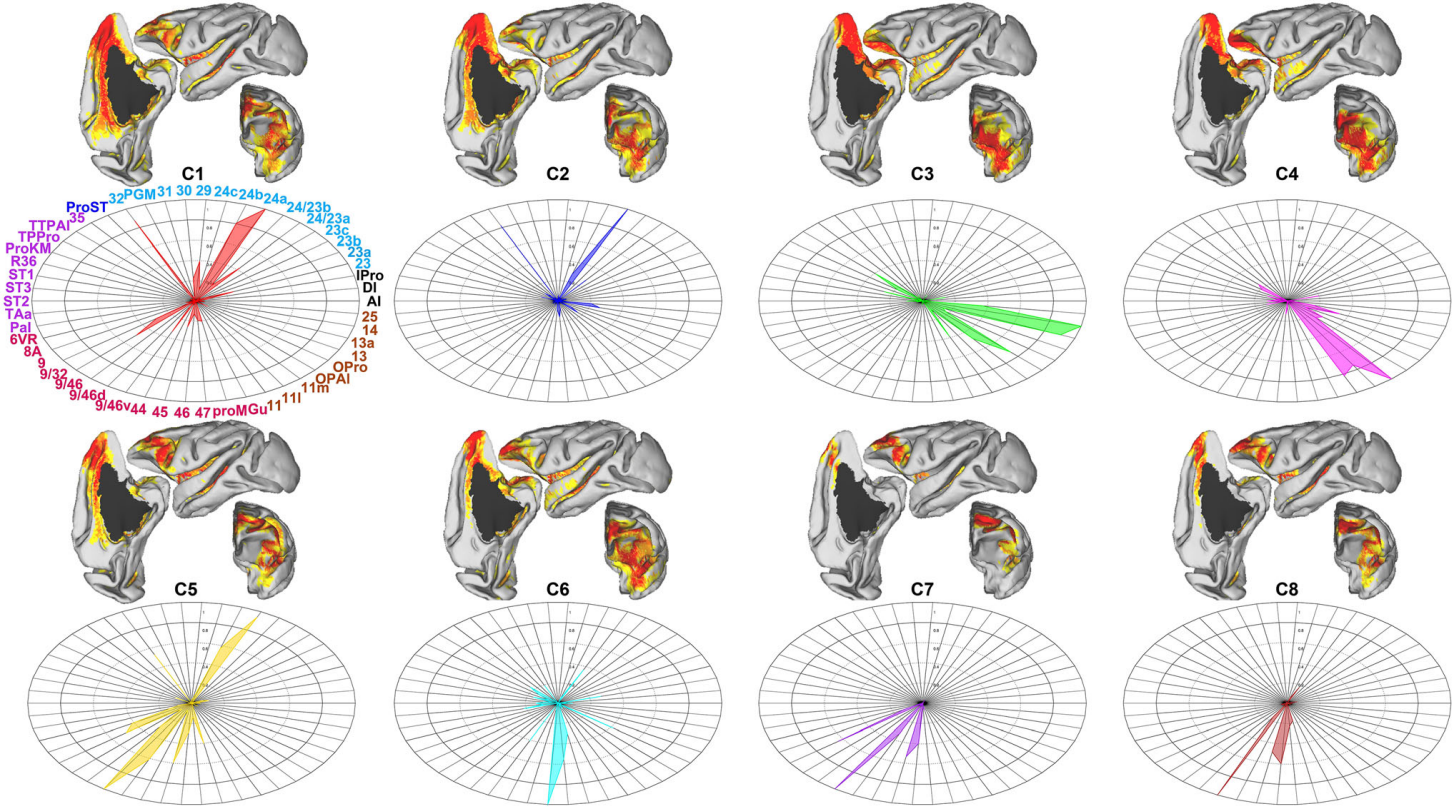
Anatomical connectivity patterns between each subarea and cortical structures (left hemisphere). The connectivity of each cluster yielded by tractography-based parcellation shown in the F99 surface using Caret helps to qualitatively identify differential connections. Anatomical connectivity fingerprints quantitatively identify the differences of the connectivity patterns between each subarea and the cortical structures. For the fingerprints, we classified the connected brain regions on the periphery of the ellipse based on the different brain structure to which they belong, and display them using different color fonts (starting from area AI, and anticlockwise, the regions with different color fonts represent the insular, cingulate, occipital, temporal, frontal, and orbitofrontal cortices). Each subarea is named C1, C2, …, C8.

**Fig. 5:**
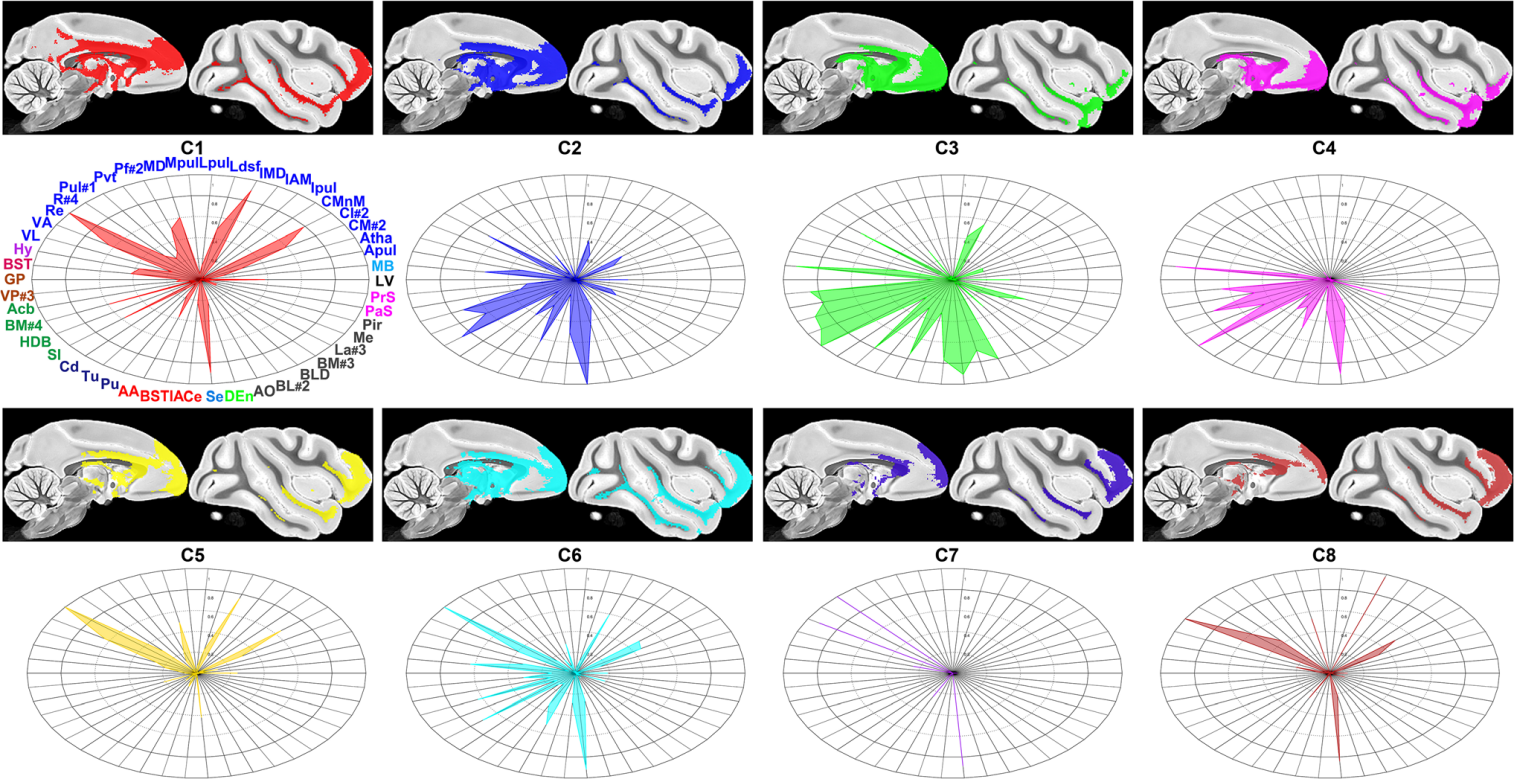
Anatomical connectivity patterns between each subarea and subcortical structures (left hemisphere). Population maps of the whole brain anatomical connectivity patterns shown in CIVM space using MRIcron help to qualitatively identify differential connections, and the connection pattern of each area is colored differently. Anatomical connectivity fingerprints quantitatively identify the differences of the connectivity patterns between each subarea and the subcortical structures. For the fingerprints, we classified the connected regions on the periphery of the ellipse based on the different structure to which they belong, and display them using different color fonts (starting from area LV, and anticlockwise, the brain regions with different color fonts belong to the lateral ventricles, midbrain, hypothalamus, central subpallium, pallium, paraseptal subpallium, striatum, subpallial amygdala, subpallial septum, lateral pallium, ventral pallium, and medial pallium). Each subarea is named C1, C2, …, C8.

Furthermore, the anatomical connectivity patterns of each subregion were obtained from the whole-brain probabilistic tractography in native diffusion space by estimating the fiber orientations for each voxel. To minimize the effects of inter-individual variations, the probabilistic patterns of the fiber tracts were then transformed into CIVM space; then an averaged fiber tract map was calculated for each subdivision and displayed in the F99 surface. The anatomical connectivity fingerprints between the subdivisions and other brain structure areas of the CIVM atlas could identify the connectivity differences for each subarea (see Fig. 4, Fig. 5, S2, and S3 for details).

#### Cluster C1

C1 was located in the medial part of the FPC around the rostral tip of the anterior cingulate cortex (ACC), following a medial-to-lateral direction, gradually along the dorsal surface of area 32, then in front of areas 9/32 and 32. Following an anterior-to-posterior direction, with the extension of the ros, C1 extended dorsally to the cgs. Above it was Cluster C5, and its dorsal extent was limited by Cluster C8. Its ventral border was delimited by Cluster C2 just above the ros, while ventrally it was anteriorly delimited by Cluster C6. This subdivision also encompassed part of the anterior bank of the cgs. Tractography samples seeded from Cluster C1 to the cortex were mostly distributed in the medial frontal cortex, the adjacent ACC, and the posterior cingulate cortex, including areas 32, 24a, 24b, 9/32, 24/23a, 29, and 10M. At a longer distance, it connected with areas 31, 23, PGM, ProST, TTPAl, and TPPro. In the subcortical results, area C1 showed a stronger connection with Re, IMD, Cl#2, and Se.

#### Cluster C2

C2 was located in the medial part of the FPC just below Cluster C1. It lay anterior to area 32, followed a medial-to-lateral direction immediately in front of the rostral cgs and also encompassed part of the rostral bank of the cgs. In its dorsal rostral part, it extended to the most posterior part of Cluster C6. Its ventral extent was limited by Cluster C3, which was located in the orbital FPC. In the coronal plane, Cluster 2 was above the smooth extension line of the ps. The connectivity of Cluster C2 was predominantly with the medial frontal cortex and part of the lateral frontal lobe, including areas 24a, 32, the most anterior FPC, 24b, 24/23a, 14, and 10M. In addition, the subcortical structures, including Se, SI, BM#4, AA, and Re, shared a stronger connectivity seeded from area C2.

#### Cluster C3

C3 covered the medial orbital part of the FPC. Following a medial-to-lateral direction, its dorsal border was delimited by Clusters C2 and C6 and gradually continued along the ventral area of Cluster C4. Along the lateral extended direction, the morbs separated Cluster C3 from Cluster C4. C3 was heavily connected with the orbitofrontal cortex, temporal cortex, and LV, including areas 14, OPAl, 13a, and the anterior and ventral parts of area 10. This cluster had strong connections with the orbital periallocortex, the rostral part of area TL (area 36R), and the temporopolar periallocortex. Compared with the connection strength with subcortical structures, Cluster C3 shared stronger connections with SI, HDB, Se, and BST.

#### Cluster C4

A distinct cluster, C4, occupied the lateral orbital part of the FPC. In the coronal plane, following an anterior-to-posterior direction, it was below Cluster C6 and its medial border was delimited by Cluster C3, gradually disappearing with Cluster C6. Its lateral part was below the ps and extended medially above the ps near the interface of ps and ros from the sagittal section followed by its extension to the anterior of area 11 in the lateral orbitofrontal cortex. The connectivity of Cluster C4 was similar to that of cluster C3. The difference was that the former had stronger connections with the lateral and orbital frontal cortex than the latter. For instance, stronger connections with Cluster C4 were detected throughout area 11, the ventral part of area 10, and the orbital proisocortex. In contrast, the connections with subcortical structures, including SI, Se, BM#4, and BST, were similar but fewer than those for Cluster C3.

#### Cluster C5

C5 covered a small region, relative to the other subregions, in the medial dorsal part of the FPC. The distribution of Cluster C5 was mainly around the white matter above the cgs from the coronal plane. It exhibited strong connectivity with the dorsolateral frontal cortex and ProM, including areas 9/46D, 24a, 9/46D, and the dorsal and medial parts of area 10. It also had a stronger connection with 6VR than the other subareas. Some subcortical structures, i.e. Re, IMD, VA, and Cl#2, had a strong connectivity with Cluster C5.

#### Cluster C6

C6, which was located in the lateral middle part of the FPC, was focused around the anterior of the aspd. It lay above the ps and a portion of this subregion extended to the medial surface. Following a medial-to-lateral direction, its superior and posterior borders were delimited by Clusters C1 and C2, then, gradually, with the disappearance of these two subdivisions, its superior border was limited by Cluster C8. This subdivision was around the rostral part of the ps and covered part of its anterior bank. Its inferior adjacent subarea was Cluster C4. Cluster C6 was mainly connected to the anterior-most frontal cortex, 46, 47, and the orbital proisocortex. With respect to the subcortical structures, it had a strong connectivity with Re, Se, SI, BM#4, and BST.

#### Cluster C7

C7 occupied the dorsal lateral part of the FPC. It was delimited posteriorly by Cluster C5 near the medial surface, gradually limited by the adjacent area 9 and covered the posterior bank of the aspd following a medial-to-lateral direction. The ventral extension was limited by Cluster C8. It was characterized by a very strong connectivity with the dorsal and lateral frontal lobe, including the adjacent areas of 10D, 9/46D, 9/46V, 45, 46, and 9. In addition, the subcortical structures R#4 and VA shared a stronger connectivity seeded from area C7.

#### Cluster C8

C8 was located in the dorsal inferior part of the lateral FPC. It covered a large part above the most anterior limb of the ps. C8 occupied a small area near the medial surface, relative to the other clusters. Following a medial-to-lateral direction, it was gradually sandwiched between C7 and C6 and extended to the superior margin of area 46D near the most lateral surface. The connectivity patterns of Clusters C7 and C8 were very similar, but the latter had stronger connections with areas 9/46V, 46, 47, and ProM.

### Similarity Analysis and Repeatability of Connected Brain Regions, and FPC Modularity Structure

**Fig. 6:**
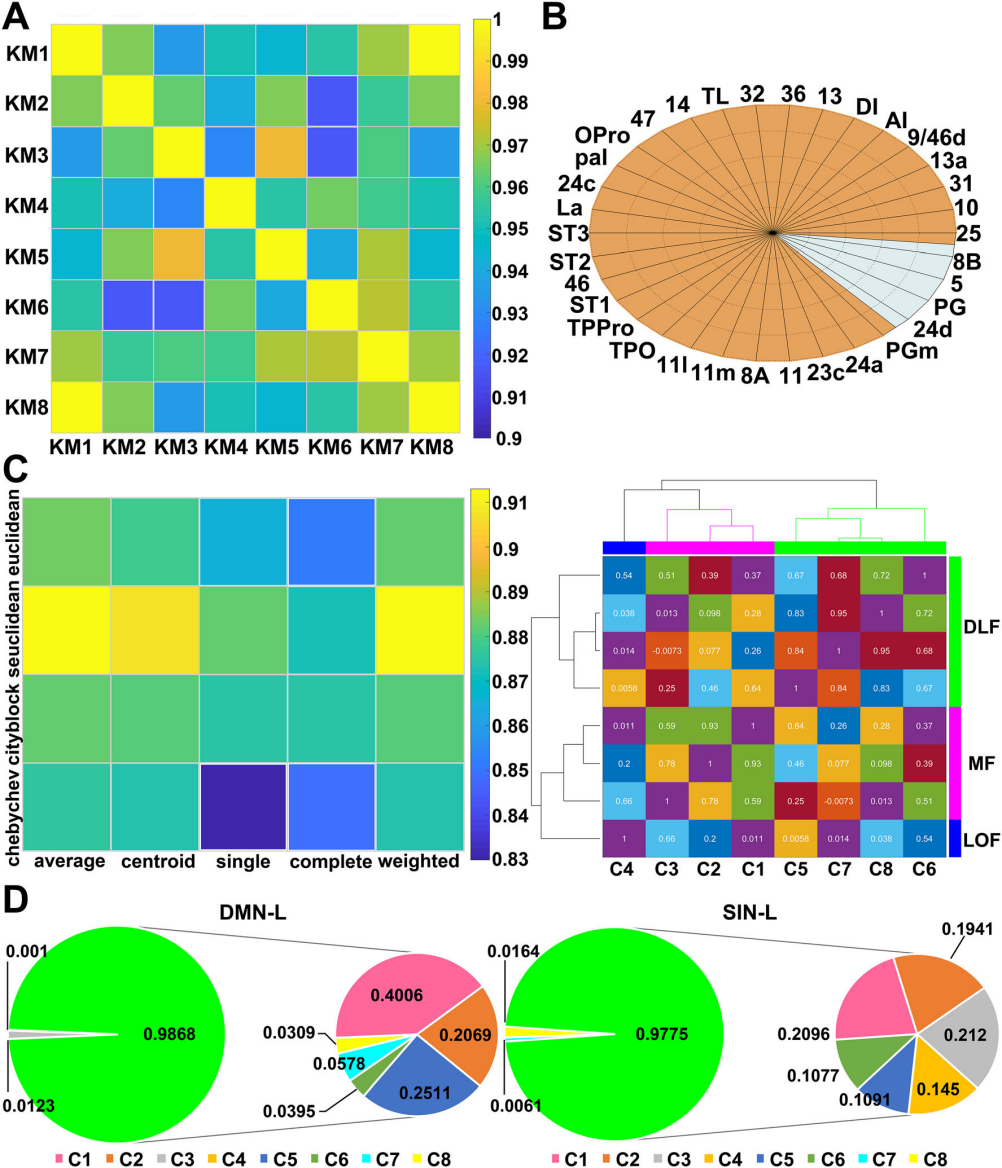
Similarity analysis and repeatability of connected brain regions, and modularity analysis (left hemisphere). **A** The connectivity similarity matrix for all the subareas across different subjects (KM1, KM2, …, KM8). **B** Consistency comparison between tracer projections of CoCoMac and the anatomical connections identified by our study. The areas around the outside edges of the ellipse are the tracer results from CoCoMac; the areas marked in orange are the anatomical connections we found, and the gray means that we did not find these connections. **C** Optimization cophenetic coefficient parameter selection, connectivity similarity matrix, and dendrogram constructed on the basis of connectivity similarity for all the clusters. **D** Diagrammatic summary of the primary connections between the subdivisions and the regions of different functional networks. The connection probabilities involved in different functional networks for each subarea are normalized in this display. Each block of the pie chart represents the anatomical connection after normalization between each subarea and the regions of different functional networks. The green circles on the left represent the sum of the primary connections on the right.

After the connection differences between subdivisions were determined, the inter-individual correlation index revealed a high level of connection similarity for the eight clusters (Fig. 6A and S4A), which suggests that the connectivities of parcellation results are very consistent among the eight subjects. Using the CoCoMac data, we identified the areas derived from our connectivity data and theirs that were derived from the axonal tracer projections that originate in the FPC, and calculated an 88.24% coherence (Fig. 6B and S4B), which to some extent suggests repeatability of the connected areas and the reliability of our parcellation results.

Then, using the improved hierarchical clustering method, the eight subdivisions were grouped into three contiguous boundary connectivity families: medial FPC (MF; C1, C2, C3), dorsolateral FPC (DLF; C5, C6, C7, C8), and lateral orbital FPC (LOF; C4) (Fig. 6C and S4A). A dendrogram was constructed using the standardized Euclidean distance (seuclidean) and average linkage (average) method because this method led to the most faithful representation of the original distances based on their highest cophenetic coefficients (left brain, 0.92; right brain, 0.94). As a mediator of network modularity in the macaque [66], these FPC subregions connected to other regions with inter-area coordination; in detail, the MF mainly connected the “medial” brain network, the DLF connected to most of the regions of the dorsal brain network, and the LOF mainly connected to the orbital brain regions.

Further, based on the anatomical connections of each subarea and the modular analysis, we found that these subareas were collaboratively involved in the DMN, SIN (Fig. 6D and S4D), and metacognition network. First, previous studies have revealed that the macaque FPC is functionally correlated with the DMN [22, 67]. We found that the DLF, in conjunction with an extension to the medial part (C1, C2), had strong connection probabilities to areas of the DMN. In particular, areas C1, C5, and C7 had a strong connection with the DMN core. Second, our results revealed that the orbital and medial FPC play an important role in connectivity to the SIN, and the medial FPC showed a stronger connection than other parts of the FPC with the exclusively SIN (ESIN) [23]. The medial FPC (areas C1, C2, and C5) had a strong connection with areas 32, 10M, 24b, 9M, 44, 6VR, and 24b. Areas C3, C4, and C6 showed a strong connection with areas 14, 47, OPro, 11L, 10o, R36, ST1, ST2, TAa, TPPro, Cd. In *a*ddition, the dorsal FPC had strong connections with the regions involved in metacognition; in particular, subareas C5, C7, and C8 had a strong connection with the cortical region anterior to the pspd (aPSPD) and metamemory processing regions, including areas 9L, 9/46D, 46D, 8A, and 6VR. Besides, the regions of metacognitive performance for remote memory have mainly been identified in the dorsal frontal lobe; dorsal FPC showed strong connections with them. These dorsal FPC subareas mainly connected the memory retrieval regions, including the anterior bank of the frontal cortex (area 45B) and area 9/46V, but we found no connections between the FPC and the parietal cortex. Further, subarea C5 had a strong connection with the cortical network module of retrieval-related regions (area DI and 6VR).

## Discussion

In this study, we performed a tractography-based parcellation and divided the macaque FPC into eight subregions, and then elucidated the anatomical connectivity patterns of the macaque FPC at the subregional level, and finally explored the modularity of the eight subareas. To more fully elucidate the reasonability of our parcellation results, we compared our parcellation and anatomical connectivity results from DTI data with previous relevant studies from a variety of perspectives, including the overlap between our boundaries and those from other parcellation results and between our connection results and those that were obtained using tracer injections.

**Fig. 7:**
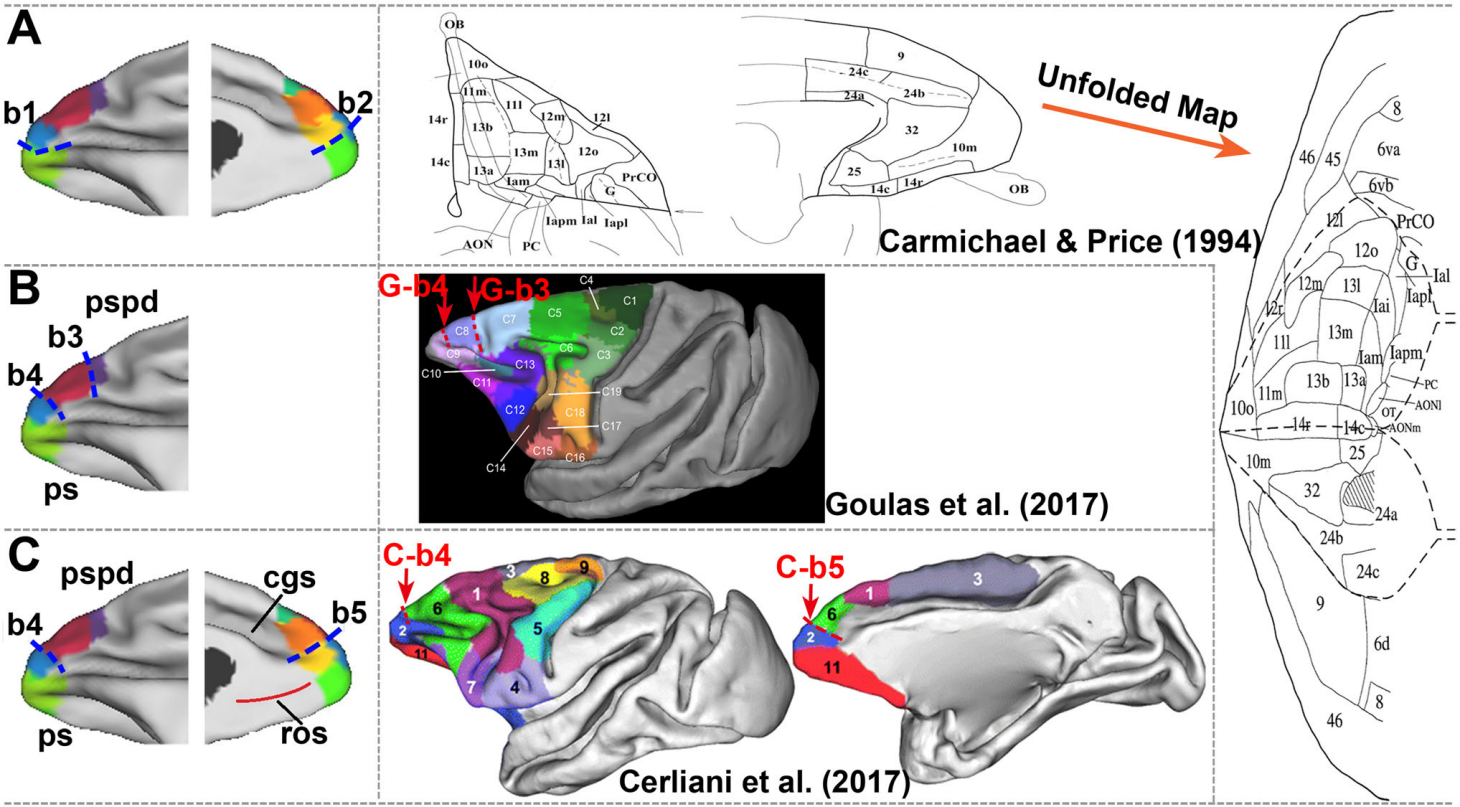
Side-by-side comparison between the results of the group-averaged, connectivity-based parcellation described in the current study (left) and the macaque maps (right) from other studies. **A** The subareas of Carmichael and Price (1994) can be further subdivided. **B** The lateral boundary b1 and medial boundary b2 distinguish the lateral subareas C4 and C6 and the medial subareas C2 and C3, respectively. Other lateral boundaries, b3 and b4, corresponding to the red boundaries of Goulas et al. (red arrow, G-b3 and G-b4), distinguish the lateral subareas C7 and C8 and subareas C8 and C6, respectively. **C** The medial boundary b5, corresponding to the medial red boundary of Cerliani *et al*. (red arrow, C-b4 and C-b5), distinguishes the medial subareas C1 and C2.

First, the eight subregions were distinguished by different boundaries, some of which coincided well with those of other parcellation studies [30, 68]. The parcellation results of the macaque FPC have varied over the past few decades. Initially, the FPC was recognized as a single area; then it was subdivided into two areas by Carmichael, Price [20], who thought that the medial part of the FPC had a homogeneous granular structure. Subsequently, however, different connections of subareas in this area were found [19, 69, 70]. Here, we found that the orbital FPC can be further subdivided into two subregions (C3 and C4) and area 10m of Carmichael, Price [20] can be subdivided into a number of subregions (Fig. 7A). In addition, the dorsolateral boundary (G-b3) and another lateral boundary (G-b4) above the rostral ps in the macaque frontal cortex in another parcellation result [68] have positions similar to the boundaries between C7 and C8 (b3) and between C8 and C6 (b4), respectively, in our parcellation results. The lateral boundary (b4) and another boundary (b5) along the anterior extension of the ros were approximatively the same in another study (C-b4 and C-b5) [30]. These findings, on the one hand, support the reliability of previous boundary parcellations. On the other hand, they imply that the CBP technique can identify even more possible subdivisions. In particular, the FPC, a thick, highly granular cortex, has gradual differences that make it difficult to further parcellate additional subareas using traditional methods [71].

Second, our anatomical connection results are in good agreement with those of the tracer injection experiments, which are the gold standard for assessing connectivity. This revealed a potential concordance of the relationship between the connectional and microstructural properties of brain regions. In addition to the consistency comparisons with CoCoMac, comparisons with other tracer experiments that have more narrowly defined injection sites well support our anatomical connection and parcellation results. It is particularly worth noting that the tracer injection results of Saleem, Miller, Price [26] provide good evidence for our segmentation results. In that study, the 10mr injection site covered approximately the same area as our dorsal subareas (C1, C2, C6, and C8), and the orbital subareas (C3 and C4) correspond to a different injection site (10o). For the cortical connections, we found rich intrinsic connections between the FPC and the prefrontal cortex (PFC) and distinct extrinsic connections with these regions outside the PFC, a finding which is consistent with the tracer projection results of Saleem *et al*. (see Table 2). The intrinsic connections between the orbital subareas and areas 11m, 12o, 12l, 10m, 10o, 46d, 32, 13b, 14r/c, and AI, as well as the extrinsic connections with areas ST1, ST2, and ST3 are consistent with the results of Saleem *et al*. In addition, the dorsal subareas have intrinsic connections with areas 46v, 45a, 45b, 8AD, 10mr, 10o, 9m, 9l, 46d, 13m, 12o, 10mc, 11m, 32, 25, AI, 13a/b, and 14r/c and extrinsic connections with areas ST1, ST2, ST3, 24a, 24b, 24c, 23, v23, and 29/30. These intrinsic and extrinsic connections were also found by Saleem *et al*. In addition, we also found no connections between the FPC and the parietal cortex, which is consistent with the conclusions of Saleem et al. [26], Petrides and Pandya [72], and Rushworth et al. [6]. More comparisons of cortical connections and subcortical connections further suggest good consistency between our anatomical connections and other tract-tracing studies (details in Table 2 and Table 3).

**Table 2:** Consistent cortical connections from our tractography compared with the data acquired in previous studies using tracer injection.

Experiments	Cases/case no.	Injection site(s)	Corresponding subregions	Projections found
Barbas and Mesulam [73]	Case V, (HRP)	Rostral principalis region (rostral 46 and 10)	C6, C8, C7	Dorsal and medial parts of the FPC, 14, 46, 12, 11, 9/46D, R36, TTPAl, ST1, ST2, ST3, TAa
Barbas and Pandya [27]	Case 1, (isotope injection)	OPro		C3, C4, C6, C7
	Case 3, (isotope injection)	14, 13		C3, C4
	Case 4, (isotope injection)	Orbital area 12		
	Case 6, (isotope injection)	46		
Barbas *et al*. [74]	Case ARb, (FB)	Medial area 10	C1, C5	9, 46, 24, 32, 12, 14, 11, 25, 8, 13, OPro, TTPAl, TPPro
Germuska *et al*. [12]	Cases BA, (BDA)		C4	ST1, ST2, TTPAl, TPPro
	Cases BC, BF, (BDA)		C7, C8	ST1, ST2, ST3
Parvizi *et al*. [75]	M1-BDA-23B, M1-FB-31, M2-BDA-23a/b, M3-BDA-29/30(23a)	23b, 31, 23a, 23b, 29/30(23a)		Dorsal and dorsomedial parts of the FPC (C1, C2, C5, C8)
Petrides and Pandya [76]	Case 4, (DY)	Lateral area 9		Dorsal subareas (C5, C7)
	Case 6, (FB)	9/46d		C1, C5
	Case 8, (FB)	Dorsal area 46 (close to FPC)		FPC
Petrides and Pandya [72]	Case 1, (isotope injection)	Area 10	FPC	9, 46, 32, 11, 13, 14, 8A, 47/12, 45, 23, 24, 25, 30, OPro, ST1, ST2, ST3, TAa, TPPro, PaI, AI, DI
	Case 2, (isotope injection)	Ventral and orbital area 10	C4	46, 10, 11, 14, 47/12, 14, 13, 32, 25, TAa
Saleem *et al*. [26]	OM19 (FB)	10o	C3, C4	47/12(12o,12l), 10mr, 10o, 46(46d), 13(13b), 10mc, 11m, 14(14r/c), 32, AI, ST1, ST2, ST3
	OM69 (FB), OM64 (FB)	10mr	C1, C2, C6, C8	46(46d, 46v, 46f), 45(45a, 45b), 8A(8Ad), 10mr, 10o, 10mc, 9 (9d, 9m), 13(13m/l, 13a, b), 47/12(12o), 11m, 14(14r/c), 32, AI, 25, ST1, ST2, ST3, 24a, 24b, 24c, 23, 29, 30

**Table 3:** Consistent subcortical connections from our tractography compared with the data acquired in previous studies using tracer injection.

Experiments	Cases/case no.	Injection site(s)	Corresponding subregions	Projections found
An *et al*. [69]	Case OM36, (BDA)	10m	C1, C2	dorsolateral midbrain PAG
	Case OM38, (BDA)	10o	C3	
	Case OM32, (FB)	Ventrolateral midbrain PAG		C1, C2, C5, C6, C7, C8
	Case OM35, (FB)	Dorsolateral midbrain PAG		
	Case OM36, (FB)	Rostral dorsolateral midbrain PAG		
	Case OM36, (CTb)	Lateral midbrain PAG		
Ferry *et al*. [77]	Case OM38, (BDA)	10m	C1, C2	Cd, Acb
	Case OM38, (BDA)	10o	C3, C4	Cd, Acb, Pu
Ghashghaei *et al*. [78]	Case BD_R_BDA Case BD_L_BDA	BM#3, BL#2, BLD, Me, Ce		C1, C2, C3, C4, C6
Hsu and Price [79]	Case OM74, (FR) Case OM66, (FR)	10m	C1, C3	MITN, Re, CM#2, CMnM, Cl#2
Ongur *et al*. [70]	Case OM26, (FB)	Lateral hypothalamus		FPC (10m, 10o)
	Case OM27, (FB)	Ventromedial hypothalamic nucleus		
	Case OM37, (FB)	Anterior hypothalamus		
Petrides and Pandya [72]	Case 1, (isotope injection)	Area 10	FPC	Lv, Cd, Pu, thalamus, Pul#1, IAM, IMD, Hy, amygdala, BL#2, BLD, BM#3, hypothalamus
Rempel-Clower and Barbas [80]	Case SF, (HRP)	Dorsal area 10	C6, C8	hypothalamus
Romanski *et al*. [81]	Case Fig. 7C, (WGA-HRP)	FPC	C1, C2, C4, C6	MPul, Pul#1
	Case 1, (WGA-HRP)	Medial pulvinar		C1, C4
	Case 2, (WGA-HRP)	Central/lateral PM		C4
	Case 3, (WGA-HRP)	Medial region of the PM (intruded on caudal, medial regions of the mediodorsal nucleus)		C1, C3,
Cho *et al*. [82]	Cases J12FR, J12LY, J16LY, J8LY, J12FS	BM#4		C1 ,C2, C3, C4

*FB* fast blue, *DY* diamidino yellow, *HRP* horseradish peroxidase, *BDA* biotinylated dextran amine, *CTb* cholera toxin subunit B, *WGA-HRP* wheat germ agglutinin-horseradish peroxidase, *FR* fluoro-ruby, *LY* Lucifer yellow, *FS* fluorescein.

In addition, the topological modules of brain networks often consist of anatomically neighboring cortical areas, and exploring the brain modular structure can heuristically facilitate functional studies of localized areas and help to understand brain mechanisms [62, 63, 83]. Our results from the hierarchical clustering for the connectivity-based parcellation revealed that the different subregions collaborate to connect different functional networks.

We found that the orbital FPC connected with most of the regions of the “orbital” prefrontal network; the medial FPC mainly linked to the “medial” prefrontal network, which is consistent with the descriptions of previous network partitions [84]. Subsequently, eight subareas are collaboratively involved in the DMN, SIN, and metacognition network. The FPC has a functional involvement with the SIN [23, 85], and the medial frontal regions around the rostral tip of the ACC show significant activity during interactive social communication [86]. Correspondingly, we found that area C1, which is located around the rostral tip of the ACC, had a strong connection with the regions of the SIN. The theory of mind (ToM) and the DMN intersectional regions in the human brain have a fairly plausible homology and locations similar to the ESIN regions of the macaque brain. These macaque areas are in locations similar to the DMN and ToM intersectional regions in humans and share anatomical features with the human ToM and ESIN; these findings are confirmed by our results. With respect to the third function, metacognition, in macaque monkeys only the bilateral FPCs are enlisted for the metacognitive evaluation of non-experienced items, the dorsal FPC is only significantly correlated with metacognitive performance with respect to non-experienced items and serves as the neural substrate for awareness of one’s own ignorance in macaques [24]. The FPC is functionally connected with the aPSPD, which is essential for the metacognitive judgment of remote memory; in particular, there is a strong resting-state functional connectivity with area 9 that is related to metamemory processing [58]. Here, we found anatomical connections between the FPC and the aPSPD with particularly strong connections with area 9. For retrieval of remote memory, the FPC may be involved in metacognitive processing. Anatomical connections between the FPC and area 9 also suggested that these two regions work cooperatively to support metacognitive judgments in ecological situations. These findings suggested a consistent relationship between functional activation and connectivity fingerprints [87]. Previous studies have reported that the corresponding memory-related regions between humans and macaques have not been established. Consistent with the previous studies [6, 26, 72], we found no connections between the FPC and the parietal regions. Notably, the medial subareas C1 and C2 had rich connections with other regions of different functional networks, a finding which suggests that these two subareas play an important role in coordinating other subareas to participate in different network functions.

To sum up, the above comparative findings validate the reliability of our parcellation results and indicate that anatomical connections and tracer-injection studies provide consistent results. We need to mention that, although different studies [30, 68] have often disagreed about the definition of the borders of the subareas in the macaque frontal cortex, if a boundary near a similar position was found in other studies, we were more convinced of its authenticity. The anatomical connections estimated from diffusion tractography may susceptible to false positives (the tracking of pseudo-pathways) and false negatives (the inability to track pathways that have been found), and do not furnish the level of detail of the gold standard based on invasive tract-tracing techniques, but DTI has proven to be an indispensable method and can offer invaluable insights for neuroscience [88] and neuroanatomy [89, 90], including the discovery of new pathways [31], the description of whole-brain connectivity information [32], and the refinement of brain regions [28].

The methods used in the current study are discussed below, along with their advantages, disadvantages, and problems of validation. First, previous studies have suggested that the CBP method can yield more fine-grained parcellations than traditional cytoarchitectonic mapping, and compared with other neuroimaging methods, it has the pivotal strength to actually map distinct brain regions without sample size restriction [33, 91]. Second, to some extent, challenges were raised in CBP studies because of the inter-individual variability, which made it difficult to relate the anatomical connectivity patterns of a region to its functional roles [29]. Third, in this study, the efficiency of the parcellation framework based on CBP and the parameters for parcellation have been validated by many studies [33, 43, 48, 92]. In general, it is worth noting that all the parameters must be reasonable, which means that they cannot be uncommon extreme values, otherwise wrong results will be tracked. One of the effective verification methods is to compare the results of the anatomical connections with those of tracer injection [93]. There are many parameters in tractography, and all of these affect the results of fiber tracking to different degrees, more or less (i.e., number of samples, distance correction, step length, curvature, exclusion mask, track style, number of steps per sample …). In particular, some studies have reported the effects of these parameters [94–99]. However, in the current study, we mainly set two parameters (number of samples = 15000; step size = 0.2 mm), other parameters are based on the default values. All these parameters were based on previous studies [33, 42] and the official instructions of FSL. Tournier *et al*. have revealed that the dispersion in tractography is dependent on the step size; small step sizes reduce the spread of probabilistic tracking results [98]. Therefore, to explore the sensitivity of the parcellation results to the number of samples, here we used different samples of tractography and carried out repetitive parcellation experiments on the macaque FPC (left brain, see details in supplementary materials; the details of the experiment and the stability validation of the parcellation method have also been described in our previous study [42]). In addition, for CoCoMac, there are still some challenges in automatically extracting data from published studies [100]. The results of tracer experiments such as those obtained from the CoCoMac database or other databases [101] are not only limited to invasive approaches to some extent, but also limited to the number of samples and lack of consideration of individual variation. The data may also miss tiny pathways and produce slightly different projections caused by individual differences, so we agree that the combination of tractography and the tracer injection results is an effective and complementary way to assess brain connectivity, which is crucial for accurately mapping structural connectivity [102]. Advances in MRI have made it increasingly feasible to calculate their connections [93], and DTI tractography is capable of providing inter-regional connectivity comparable to neuroanatomical connectivity [103]. In the current study, the consistency of the connectivity comparison with other relevant studies increases the confidence in the structural connectivity of the macaque FPC and is important for studying FPC-related networks of brain functions and their disorders [104, 105]. In future, we plan to conduct a tracer injection based on the parcellation results in the current study to explore the detailed connectivity of each subregion by a quantitative cytoarchitectonic analysis and evaluate the degree of consistency between anatomical connections and tracer injections in the same subjects. Furthermore, we have released the detailed parcellation pipeline and will then apply it to the whole macaque brain to obtain a fine-grained macaque brain atlas.

Besides, compared with other methods, the framework of CBP provided in the current study not only inherited the advantages of other classical CBP methods [48, 50] but also improved them in selecting the optimal clustering scheme and making them more adaptable to non-human primates [42]. The results of the modularity could heuristically facilitate functional studies of localized areas and exploration of the connectivity patterns of different functional networks is important for understanding brain mechanisms and evolution. Furthermore, based on previous studies [68], our proposed hierarchical clustering algorithm can automatically select the best clustering parameters to generate an optimal clustering result by calculating and comparing all the cophenetic correlation coefficients, which is helpful for users to group the clusters into different broad connectivity families.

## Conclusions

In the present study, we used a CBP scheme for the macaque FPC and divided it into eight distinct subareas. As a powerful analytical framework, CBP not only reveals the spatial distribution of cytoarchitectural boundaries but also provides supplementary information related to the organization of anatomical and different functional networks among brain regions.

Furthermore, by using a hierarchical clustering algorithm, we identified the modularity of the bilateral FPC and found synergy related to the DMN, SIN, and metacognition network among the subdivisions. We hope that all of the above information is helpful for understanding the anatomy and circuitry of related regions and can facilitate the use of available knowledge in FPC-related clinical research, especially in understanding the dysfunctions caused by complex diseases.

## Electronic supplementary material

Below is the link to the electronic supplementary material.

Supplementary material 1 (PDF 1260 kb)

## Abbreviations of the Brain Regions

10: Area 10 of cortex
11: Area 11 of cortex
13: Area 13 of cortex
23: Area 23 of cortex
25: Area 25 of cortex
30: Area 30 of cortex
31: Area 31 of cortex
32: Area 32 of cortex
35: Area 35 of cortex
44: Area 44 of cortex
10471: Area 9/32 of cortex
15584: Area 9/46 of cortex
10D: Area 10 of cortex, dorsal part
10M: Area 10 of cortex, medial part
10V: Area 10 of cortex, ventral part
11L: Area 11 of cortex, lateral part
11m: Area 11 of cortex, medial part
13a: Area 13a of cortex
13L: Area 13 of cortex, lateral part
13M: Area 13 of cortex, medial part
14M: Area 14 of cortex, medial part
14o: Area 14o
23a: Area 23a of cortex
23b: Area 23b of cortex
23c: Area 23c of cortex
24/23a: Area 24/23a of cortex
24/23b: Area 24/23b of cortex
24a: Area 24a of cortex
24b: Area 24b of cortex
24c: Area 24c of cortex
29a: Area 29a of cortex
45A: Area 45A of cortex
45B: Area 45B of cortex
46D: Area 46D of cortex
46V: Area 46V of cortex
47(12): Area 47 (old 12) of cortex
47(12)L: Area 47 (old 12) of cortex, lateral part
47(12)O: Area 47 (old 12) of cortex, orbital part
6VR(F5): Area 6 of cortex, rostral ventral part
8AD: Area 8 of cortex, anterodorsal part
8AV: Area 8 of cortex, anteroventral part
9/46D: Area 9/46 of cortex, dorsal part
9/46V: Area 9/46 of cortex, ventral part
9L: Area 9 of cortex, lateral part
9M: Area 9 of cortex, medial part
AA: Anterior amygdaloid area
Acb: Accumbens nucleus
AI: Agranular insular cortex
AO: Anterior olfactory nucleus
Apul: Anterior pulvinar
Atha: Anterior thalamic nucleus
BL#2: Basolateral amygdaloid nucleus
BLD: Basolateral amygdaloid nucleus, dorsal part
BM#3: Basomedial amygdaloid nucleus
BM#4: Basal nucleus (Meynert)
BST: Bed nucleus of the stria terminalis central division
BSTIA: Bed nucleus of the stria terminalis intraamygdaloid division
Cd: Caudate nucleus
Ce: Central amygdaloid nucleus
Cl#2: Centrolateral thalamic nucleus
CM#2: Central medial thalamic nucleus
CMnM: Centromedial thalamic nucleus, medial part
Den: Dorsal endopiriform nucleus
DI: Dysgranular insular cortex
GP: Globus pallidus
Gu: Gustatory cortex
HDB: Nucleus of the horizontal limb of the diagonal band
Hy: Hypothalamus
IAM: Interanteromedial thalamic nucleus
IMD: Intermediodorsal thalamic nucleus
IPro: Insular proisocortex
Ipul: Inferior pulvinar
La#3: Lateral amygdaloid nucleus
Ldsf: Lateral dorsal thalamic nucleus, superficial part
Lpul: Lateral pulvinar
LV: Lateral ventricles
MB: Midbrain
MD: Mediodorsal thalamic nucleus
Me: Medial amygdaloid nucleus
MPul: Medial pulvinar
OPAl: Orbital periallocortex
OPro: Orbital proisocortex
PaIL: Parainsular cortex
PaS: Parasubiculum
Pf#2: Parafascicular thalamic nucleus
PGM: Area PGM/31 of cortex
Pir: Piriform cortex
ProKM: Prokoniocortex, medial part
ProM: Area ProM (promotor)
ProST: Prostriate area
PrS: Presubiculum
Pu: Putamen
Pul#1: Pulvinar nuclei
Pvt: Paraventricular thalamus
R#4: Reticular thalamic nucleus
R36: The perirhinal cortex
Re: Reuniens thalamic nucleus
Se: Septum
SI: Substantia innominata
ST1: Superior temporal sulcus area 1
ST2: Superior temporal sulcus area
ST3: Superior temporal sulcus area 3
TAa: Temporal area TAa
TPPro: Temporopolar proisocortex
TTPAl: Temporopolar periallocortex
Tu: Olfactory tubercle
VA: Ventral anterior thalamic nucleus
VL: Ventral lateral thalamic nucleus
VP#3: Ventral pallidum

## Other Abbreviations

ANTs: Advanced normalization tools
aspd: Anterior supraprincipal dimple
cgs: Cingulate sulcus
DMN: Default-mode network
dMRI: Diffusion magnetic resonance imaging
DTI: Diffusion tensor imaging
ESIN: Exclusively social interaction network
fMRI: Functional magnetic resonance imaging
MIPAV: Medical image processing, analysis, and visualization software
morbs: Medial orbital sulcus
MRI: Magnetic resonance imaging
PCA: Principal component analysis
ps: Principal sulcus
pspd: Posterior supraprincipal dimple
ROI: Region of interest
ros: Rostral sulcus
SIN: Social-interaction network
