# Supplementary material for: Fine-Grained Topography and Modularity of the Macaque Frontal Pole Cortex Revealed by Anatomical Connectivity Profiles
Source: Neurosci Bull. 2020 Oct 27;36(12):1454–73. doi: 10.1007/s12264-020-00589-1 (PMC7719154; doi:10.1007/s12264-020-00589-1)
Supplement: Supplementary file 1 — Supplementary material 1 (PDF 1260 kb) [file 12264_2020_589_MOESM1_ESM.pdf]

Supplementary Materials

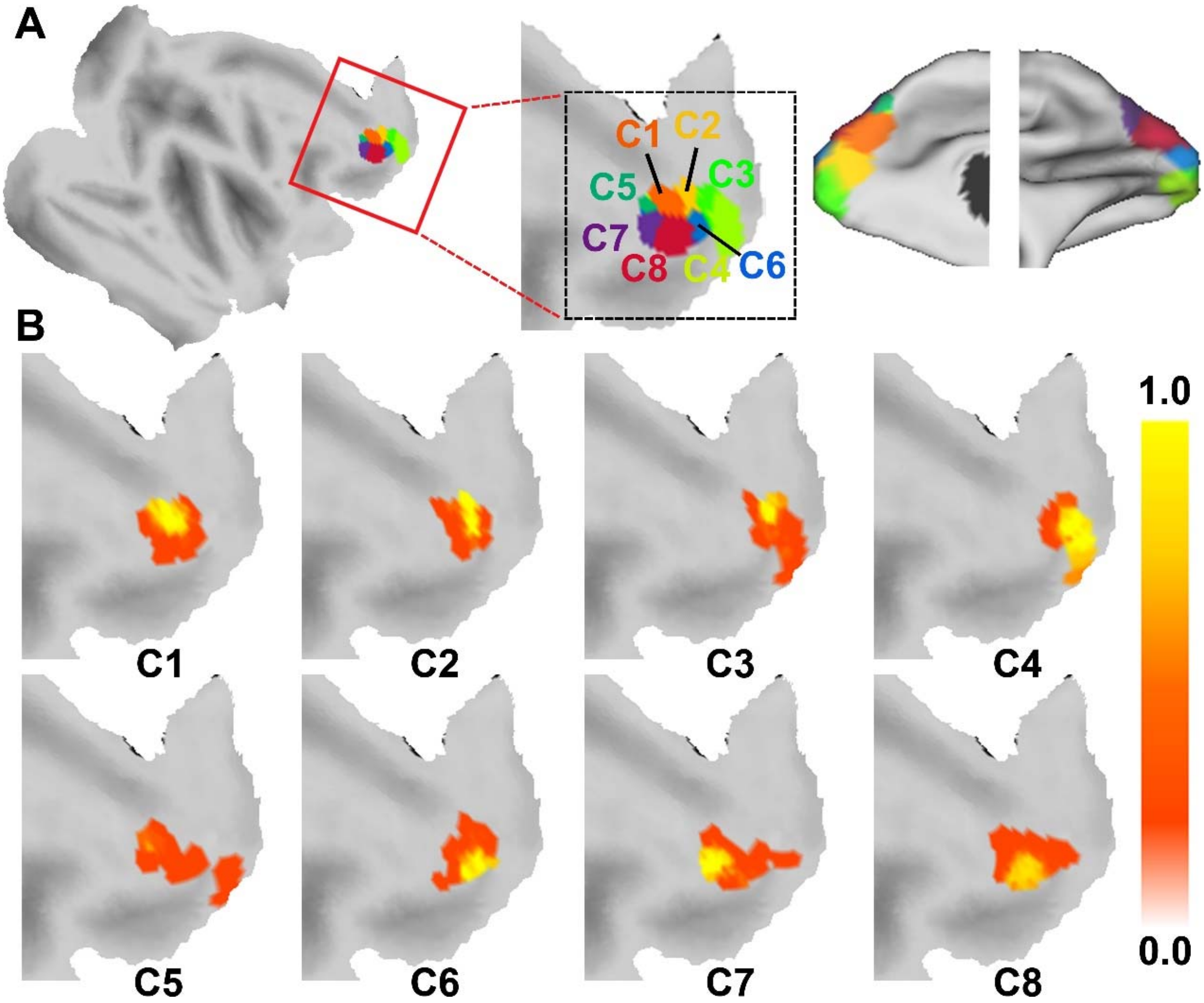

Fig. S1 Connectivity-based parcellation (right hemisphere) of the macaque FPC on F99 surfaces.

A The subdivisions are depicted on a flat surface (left) and a fiducial surface (right) of the lateral and medial views. Each subregion is coded with a unique color and named arbitrarily C1, C2, ..., C8. The probability map of each FPC subarea is shown in **B** The color bar represents the mean probability across subjects at each voxel.

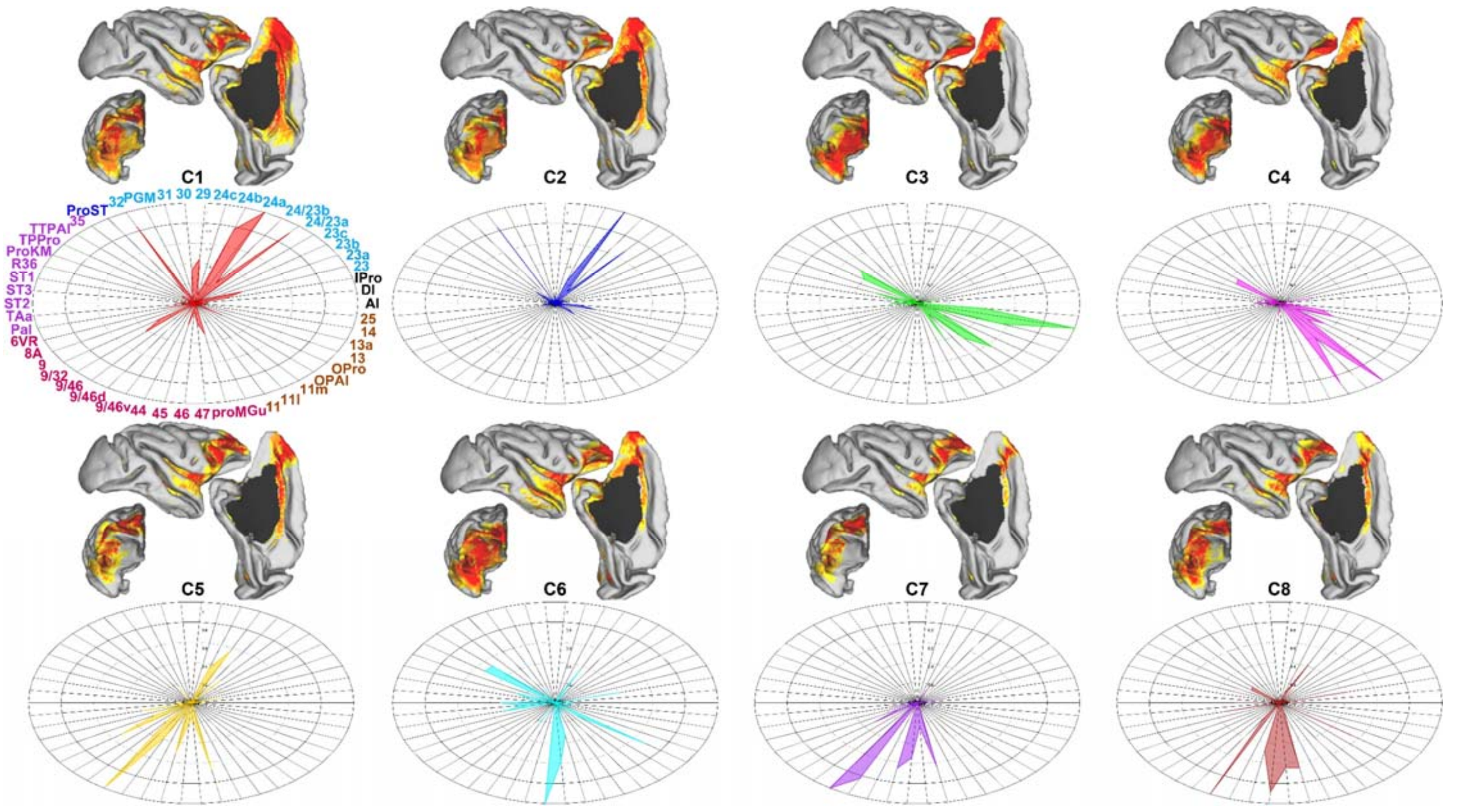

Fig. S2 Anatomical connectivity patterns between each subarea and cortical structures (right hemisphere). The connectivity of each cluster yielded by tractography-based parcellation shown on the F99 surface using Caret helps to qualitatively identify differential connections. Anatomical connectivity fingerprints quantitatively identify the differences of the connectivity patterns between each subarea and cortical structures. For the fingerprints, we classified the connected regions on the periphery of the ellipse based on the different structure to which they belong, and displayed them using different color fonts (starting from area AI, and anticlockwise, the regions with different color fonts belong to the insular cortex, cingulate cortex, occipital cortex, temporal cortex, frontal cortex, and orbitofrontal cortex). Each subarea is named **C1**, **C2**, **...**, **C8**.

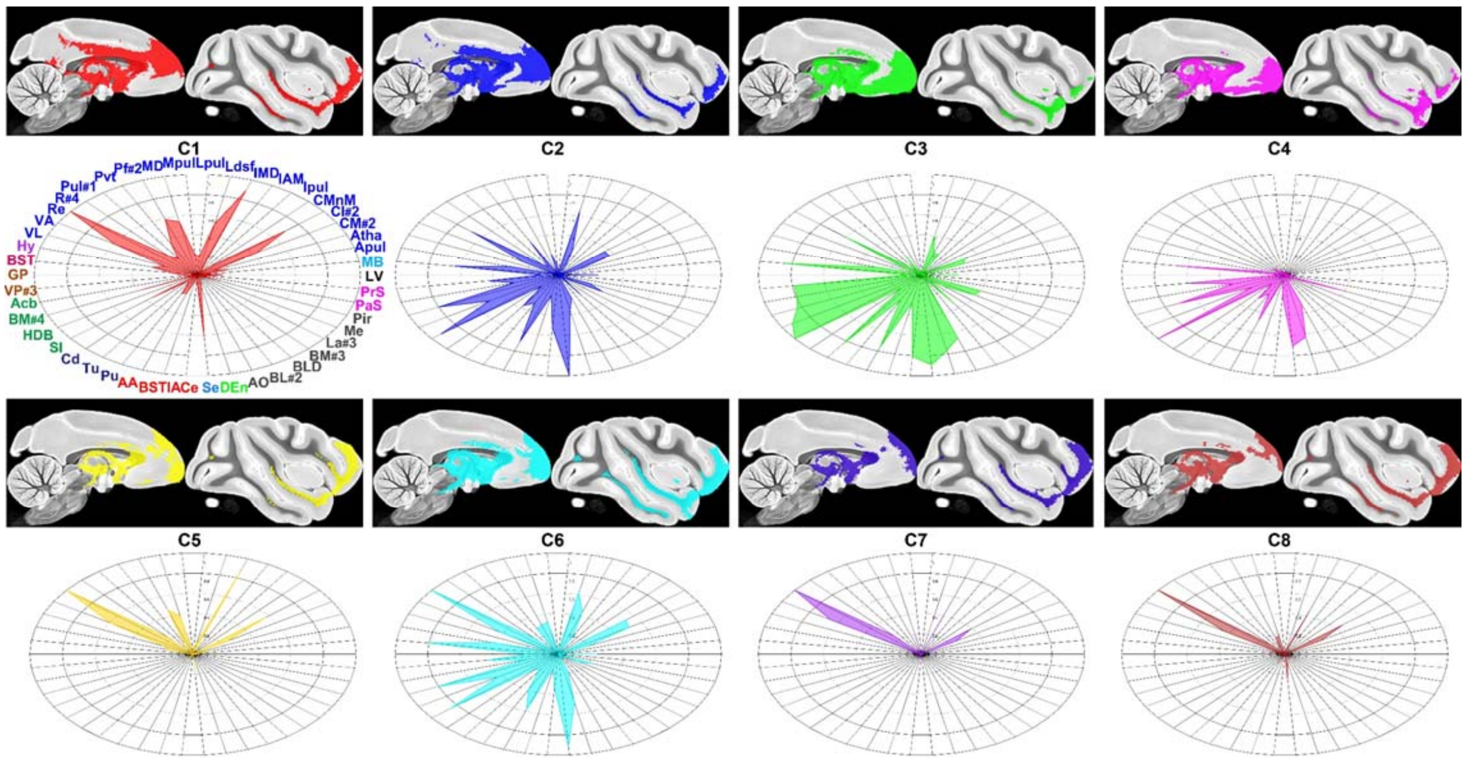

Fig.S3 Anatomical connectivity patterns between each subarea and subcortical structures (right hemisphere). Population maps of the whole brain anatomical connectivity patterns shown in CIVM space using MRIcron help to qualitatively identify differential connections, and the connection pattern of each area is colored differently. Anatomical connectivity fingerprints quantitatively identify differences in the connectivity patterns between each subarea and subcortical structures. For the fingerprints, we classified the connected regions on the periphery of the ellipse based on the different structures to which they belong, and display them using different color fonts (starting from area LV, and anticlockwise, the brain regions with different color fonts belong to the lateral ventricles, midbrain, hypothalamus, central subpallium, pallium, paraseptal subpallium, striatum, subpallial amygdala, subpallial septum, lateral pallium, ventral pallium, and medial pallium). Each subarea is named **C1**, **C2**, **...**, **C8**.

Similarity analysis and repeatability of connected brain regions, and FPC modularity structure

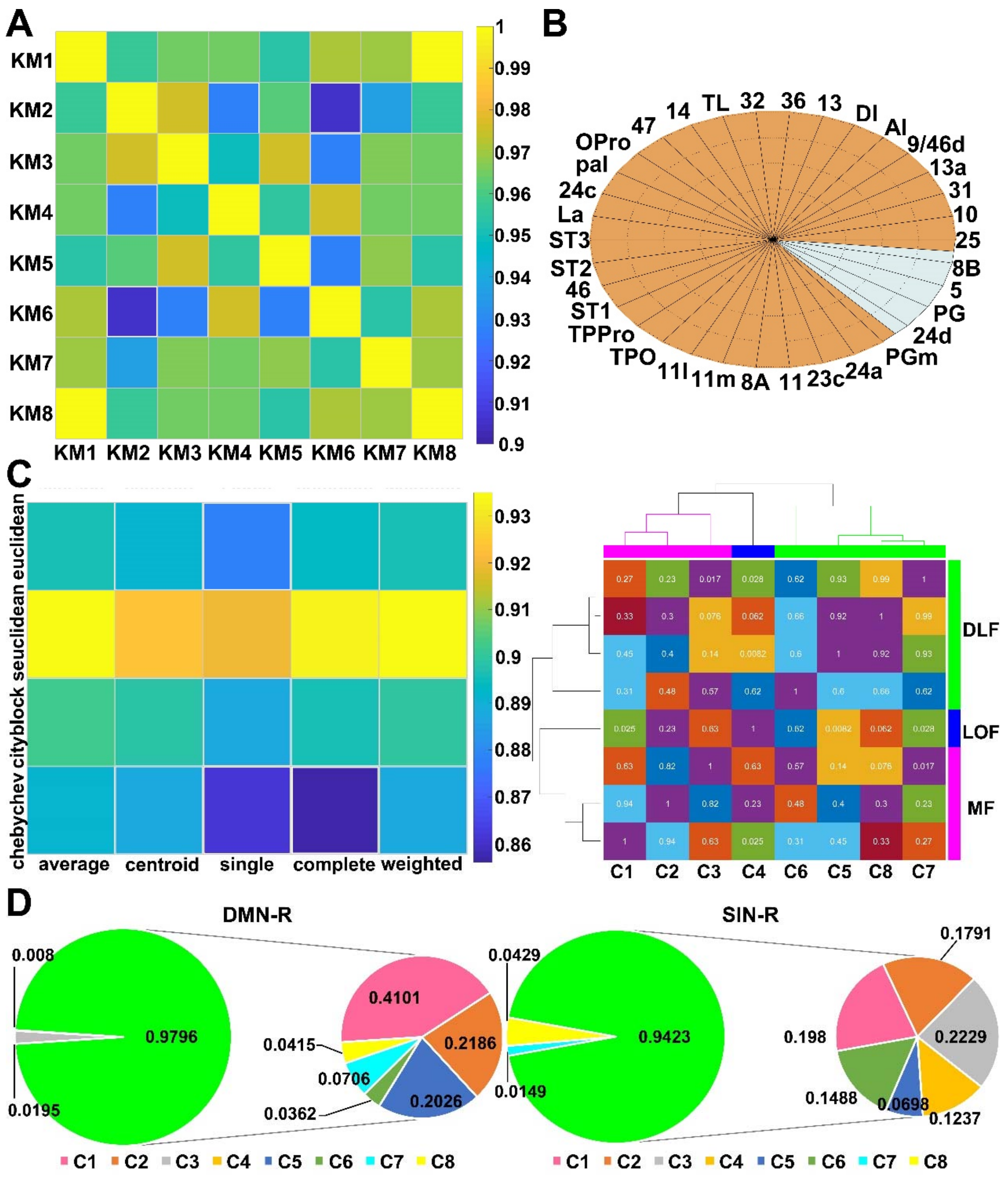

Fig. S4 Similarity analysis and repeatability of connected brain regions, and modularity analysis (right hemisphere). A Connectivity similarity matrix for all subareas across different subjects (KM1, KM2, ..., KM8). **B** Consistency comparison between tracer projections of CoCoMac and the

anatomical connections identified in our study. The areas around the outside edges of the ellipse are the tracer results from CoCoMac; the areas marked in orange are the anatomical connections we found, and gray means that we did not find these connections. **C** Optimization cophenetic coefficient parameter selection, connectivity similarity matrix, and dendrogram constructed on the basis of connectivity similarity for all clusters. **D** Diagrammatic summary of the primary connections between the subdivisions and the regions of different functional networks. The connection probabilities involved in different functional networks for each subarea are normalized in this display. Each block of the pie chart represents the connection after normalization between each subarea and the regions of different functional networks. The green circles on the left represent the sum of the primary connections on the right.

Sensitivity analysis of the parcellation results to the number of samples

To explore the sensitivity of the parcellation results to the number of samples, we parcellated the macaque FPC with three different values of streamlines/samples (15000, 13000, and 12000), and then calculated the overlap of the parcellation results. Here, we named the three parcellation results PR15, PR13, and PR12. In particular, PR15 was the current result and was regarded as the object of comparison. The other two parcellation results, PR13 and PR12, were each compared with PR15.

We calculated the number of all non-zero voxels in each post-processed maximum probability map (ppMPM), the number of overlapping voxels for the entire ppMPM, and the number of overlapping voxels of each subregion. Then we calculated the degrees of overlap were 95.12% (15000 *vs* 13000) and 91.64% (15000 *vs* 12000). Qualitatively, there was good consistency between PR15 and

PR13 (Fig. S5) and between PR15 and PR12 (Fig. S7). In addition, we calculated the degree of overlap for each subregion, for which we calculated the proportion of overlapping voxels of each subregion to itself. In particular, we calculated the number of voxels each subregion and the number of overlapping voxels of each subregion. Then we obtained eight results that represented the degree of overlap of each subregion (Figs S6 and S8, Tables S1 and S2). Besides, the distribution pattern of parcellation results was approximately consistent. All these results showed that a reasonable value of samples appears to have no clear effect on the accuracy of the parcellation results.

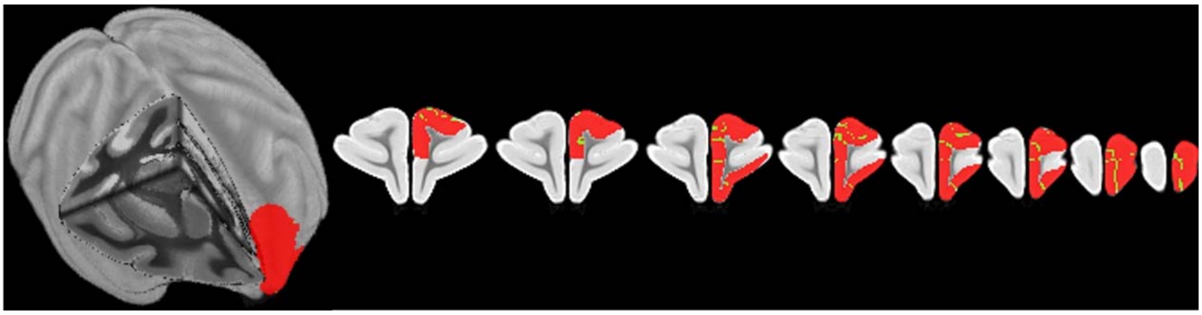

Fig. S5 Overlap between different parcellation results (15000 *vs* 13000). Left, rendered 3D diagram. Right, detailed display based on volume. Red, areas of overlap; green, difference.

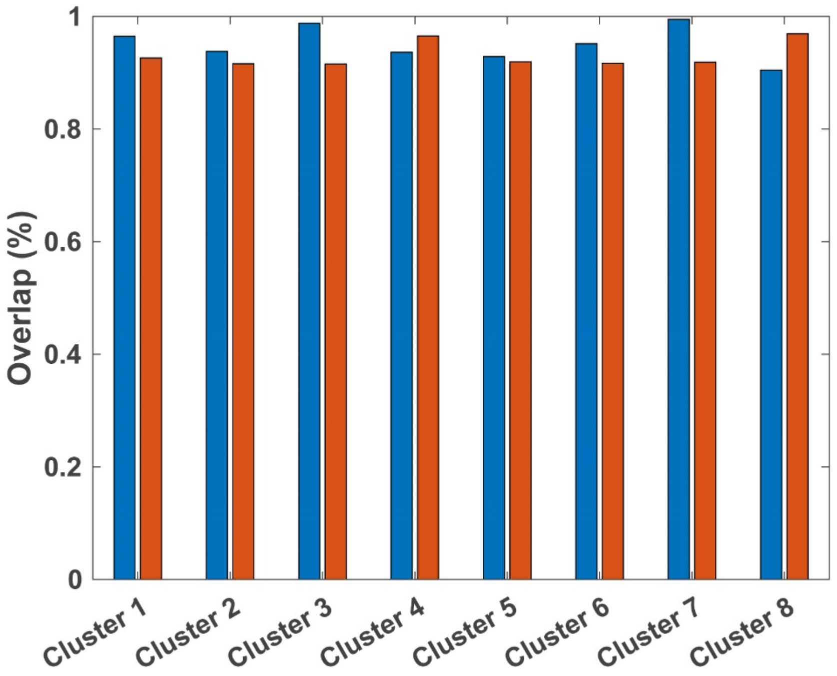

Fig. S6 Degree of overlap of each subregion/cluster (15000 *vs* 13000). Blue, parcellation results with the 15000 streamlines; orange, parcellation results with the 12000 streamlines.

Table S1. Degree of overlap of the parcellations with different parameters (15000 *vs* 13000).

Clusters	**Sample 1=15000**			**Sample 2=13000**			**Sample 1∩Sample 2**		
	Voxels	Overlap	Ratio(%)	Voxels	Overlap	Ratio(%)	Voxels	Overlap	Ratio(%)
Cluster1	1565	1510	96.49	1630	1510	92.24	-	-	-
Cluster2	1080	1013	93.80	1106	1013	91.59	-	-	-

Cluster3	1516	1498	98.81	1636	1498	91.56	-	-	-
Cluster4	1730	1620	93.64	1678	1620	96.54	-	-	-
Cluster5	675	627	92.89	682	627	91.94	-	-	-
Cluster6	1615	1537	95.17	1676	1537	91.71	-	-	-
Cluster7	1363	1356	99.49	1476	1356	91.87	-	-	-
Cluster8	1878	1699	90.47	1753	1699	96.92	-	-	-
Total	11422	11417	-	11637	11417	-	11417	10860	**95.12**

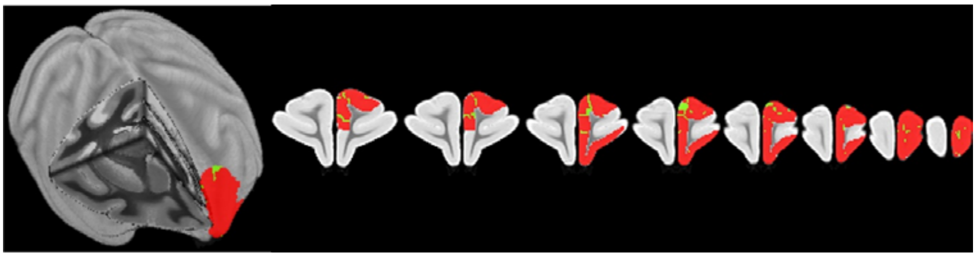

Fig. S7 Overlap between different parcellation results (15000 *vs* 12000). Left, rendered 3D diagram. Right, detailed display based on volume. Red, areas of overlap; green, difference.

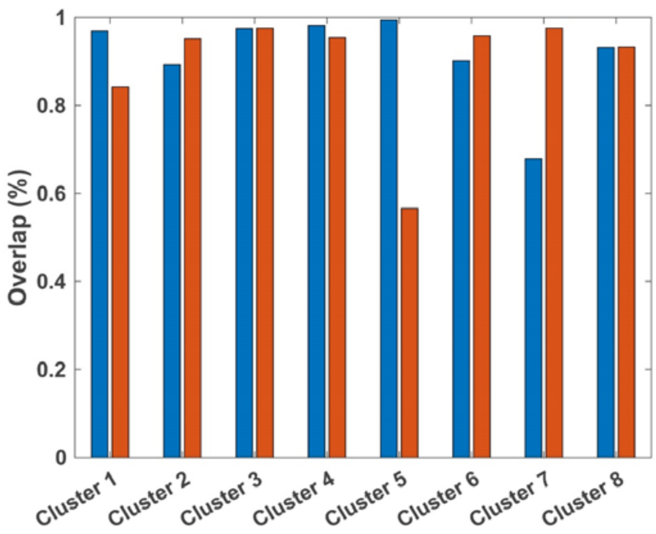

Fig. S8 Degree of overlap of each subregion/cluster (15000 *vs* 12000). Blue, parcellation results with the 15000 streamlines; orange, parcellation results with the 12000 streamlines.

Table S2. Degree of overla of the parcellations with different parameters (15000 *vs* 12000).

Clusters	**Sample 1=15000**			**Sample 2=12000**			**Sample 1∩Sample 2**		
	Voxels	Overlap	Ratio(%)	Voxels	Overlap	Ratio(%)	Voxels	Overlap	Ratio(%)
Cluster1	1565	1516	96.87	1799	1516	84.27	-	-	-
Cluster2	1080	965	89.35	1014	965	95.17	-	-	-
Cluster3	1516	1478	97.49	1515	1478	97.56	-	-	-

Cluster4	1730	1699	98.21	1780	1699	95.45	-	-	-
Cluster5	675	671	99.41	1185	671	56.62	-	-	-
Cluster6	1615	1456	90.15	1519	1456	95.85	-	-	-
Cluster7	1363	926	67.94	949	926	97.58	-	-	-
Cluster8	1878	1750	93.18	1876	1750	93.28	-	-	-
Total	11422	10461	-	11637	10461	-	11415	10461	**91.64**

We also noted that the degree of overlap of cluster 5 between PR15 and PR12 was 56.62% (PR12 *vs* PR15) and recognized that there was a difference in the position of the dorsal FPC. The reason is that this subregion was a small subdivision, and the total numbers of voxels in cluster 5 were 675 (PR15) and 1185 (PR12). This number (671) represents the intersection of cluster5 between PR15 and PR12, which means that the maximum degree of overlap was 56.96% (675/1185) and the low value of 56.62% was acceptable. Also, the distribution patterns of parcellation results were approximately consistent and the total overlap was 91.64%, which suggested good consistency.
